# Supplementary material for: Application of spectral flow cytometry for comprehensive detection of immune metabolism in patient-derived microsamples
Source: Cell Rep Methods. 2026 Mar 16;6(3):101330. doi: 10.1016/j.crmeth.2026.101330 (PMC13030956; doi:10.1016/j.crmeth.2026.101330)
Supplement: Document S1. Figures S1–S6 and Table S1 [file mmc1.pdf]

Cell Reports Methods, Volume *6*

Supplemental information

Application of spectral flow cytometry for comprehensive detection of immune metabolism in patient-derived microsamples

Yang Bai, Yuqing Wang, Yicheng Fu, Zhengyang Guo, Zhaoyuan Liang, Liu Yang, Jiawei Ribaudo, Dan Liu, Yanfang Li, Ting Zhang, Lixiang Xue, Jianling Yang, Huilin Liu, Xianlong Li, and Jie Zhang

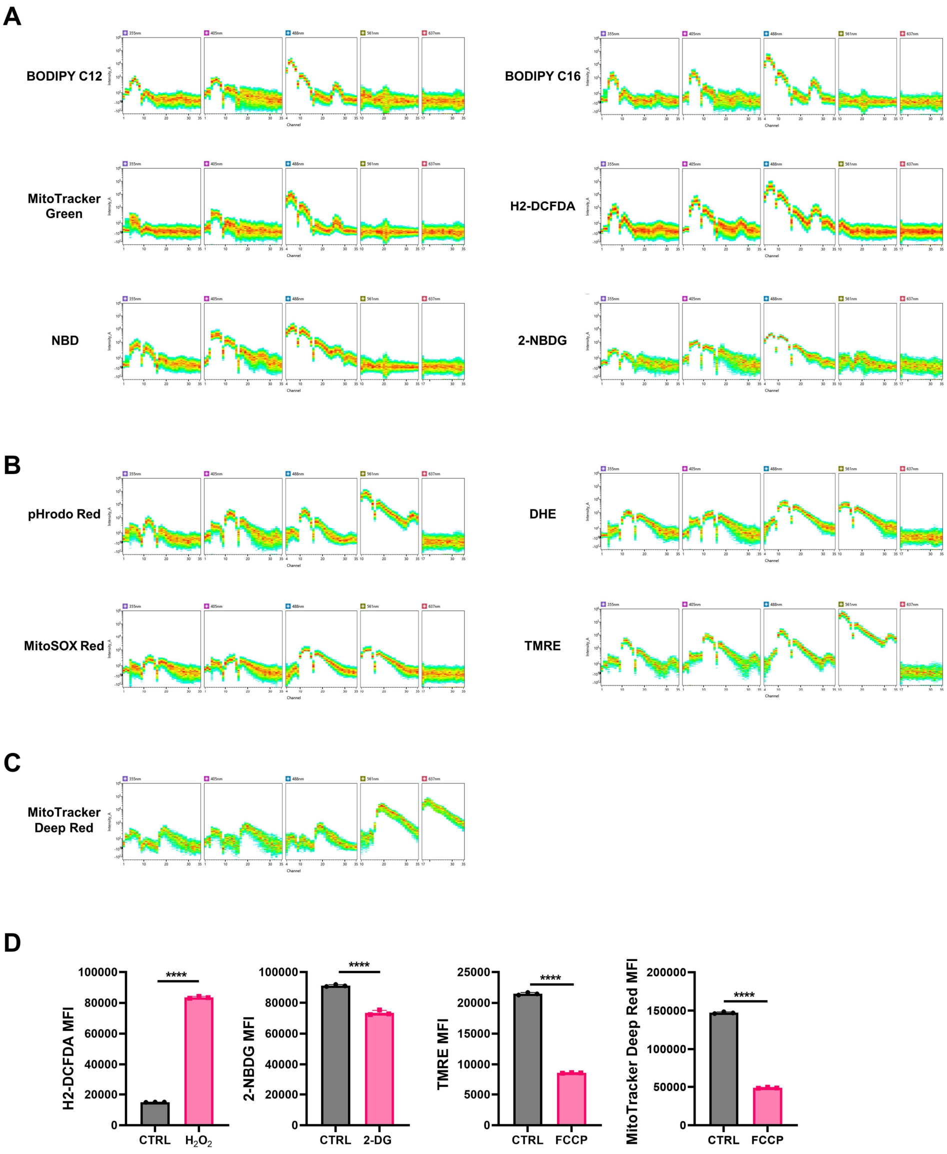

Figure S1. Establishment of an 11-probe spectral reference library, related to Figure 1 and STAR Methods.

Spectral profiles of metabolic probes for channel-specific reference generation: **(A)** FITC channel (6 probes). **(B)** PE channel (4 probes). **(C)** APC channel (1 probe). (**D**) Positive controls: H_2O_2 for H2-DCFDA. Negative controls: 2-deoxy-D-glucose (2-DG) for 2-NBDG; FCCP for TMRE and MitoTracker Deep Red (n=3). ****, $p < 0.0001$.

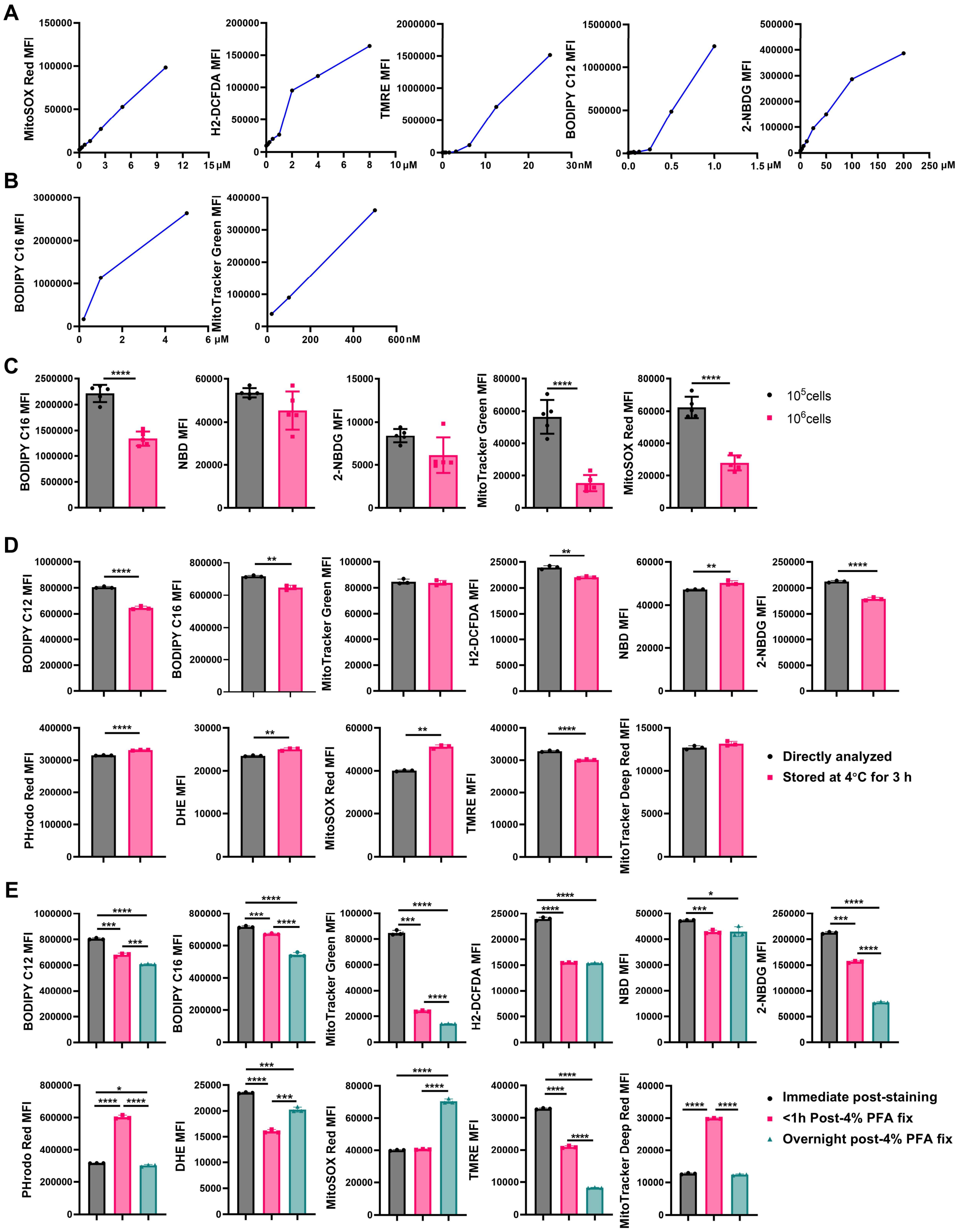

Figure S2. Characterization of metabolic probe titration and staining stability, related to Figure 1 and STAR Methods.

Titration curves of staining probes across 10 concentrations **(A)** and 3 concentrations **(B)** within instrument detection range. **(C)** Mean fluorescence intensity (MFI) of 10^5 and 10^6 A2780 cells stained with 5 distinct metabolic probes (n=5). **(D-E)** Evaluation of staining stability. A2780 cells were stained with 11 distinct metabolic probes. **(D)** MFI of cells analyzed immediately after staining was compared to that of cells stored at 4°C in the dark for 3 hours (n=3). **(E)** MFI comparison across three conditions: immediate analysis; analysis within 1 h post 4% paraformaldehyde (PFA) fixation; and analysis after overnight storage (n=3). *, $p < 0.05$; **, $p < 0.01$; ***, $p < 0.001$; ****, $p < 0.0001$.

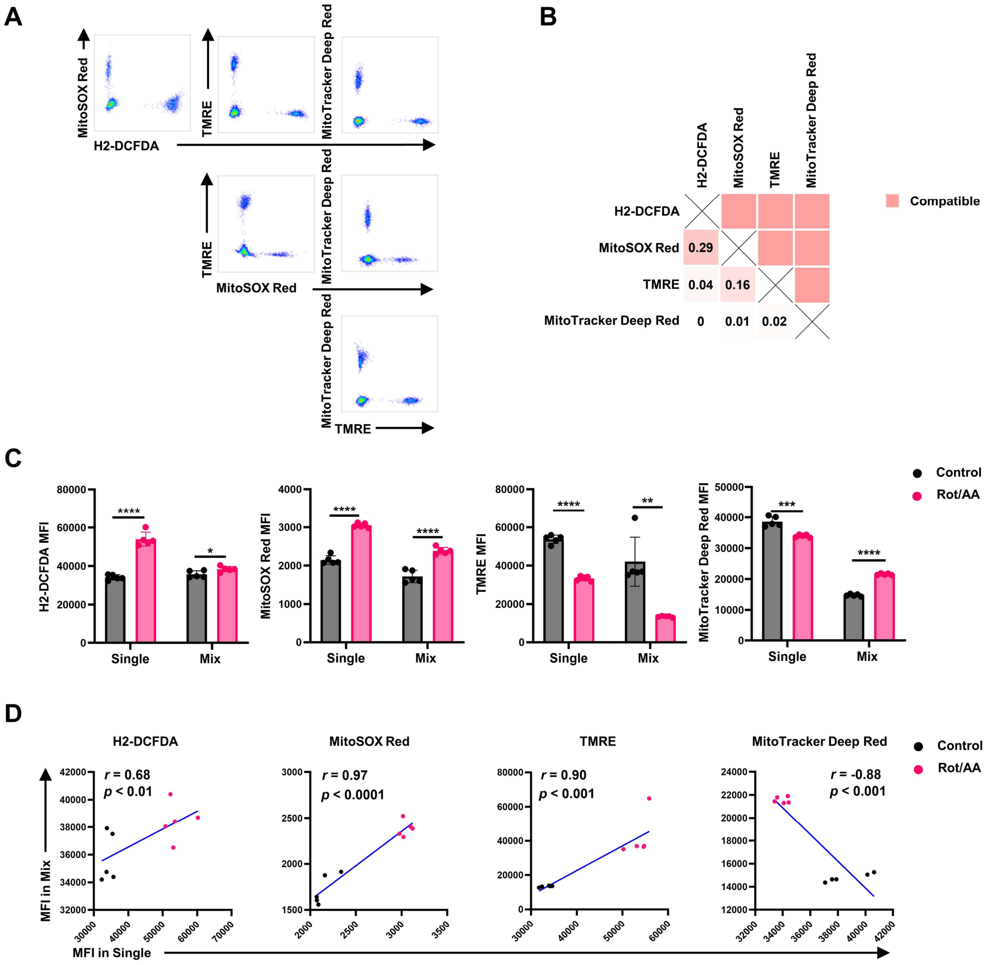

Figure S3. Validation of four metabolic probes for simultaneous assessment of mitochondrial activity and oxidative stress, related to Figure 3.

(A) Representative flow cytometry density plots demonstrating spectral resolution of co-stained probes. **(B)** Validation matrix: Lower left quadrant - Computational spectral similarity by FluoroFinder; Upper right quadrant - Empirical resolvability determination (Red: resolvable pairs). **(C)** Mean fluorescence intensity (MFI; mean ± SD) of individual probes in control versus Rotenone/Antimycin A (Rot/AA)-treated groups (n=5). **(D)** Correlation analysis of MFI between single-stain and multiplexed conditions across probes (Spearman r). *, $p < 0.05$; **, $p < 0.01$; ***, $p < 0.001$; ****, $p < 0.0001$.

A

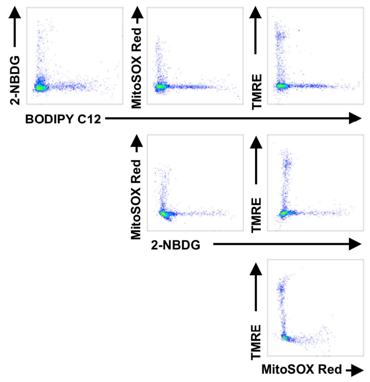

B

	BODIPY FL *	2-NBDG *	MitoSOX Red *	TMRE *	PE-Fire 640	APC-Fire 750	Brilliant Ultraviolet 737	Brilliant Ultraviolet 805	Brilliant Ultraviolet 496	Brilliant Violet 421	PE	Brilliant Violet 480	APC-Fire 810	RealBlue 744	Brilliant Violet 711	Alexa Fluor 700 *	APC	Spark NIR 685	Pacific Blue	PE-Cy7	Zombie NIR
BODIPY FL *	1.0	0.69	0.13	0.01	0.0	0.0	0.0	0.01	0.13	0.01	0.03	0.05	0.0	0.03	0.0	0.0	0.0	0.0	0.02	0.0	0.0
2-NBDG *	0.69	1.0	0.63	0.07	0.06	0.0	0.01	0.02	0.23	0.03	0.25	0.16	0.01	0.04	0.01	0.0	0.01	0.0	0.07	0.02	0.0
MitoSOX Red *	0.13	0.63	1.0	0.16	0.24	0.0	0.04	0.01	0.1	0.01	0.47	0.05	0.02	0.06	0.04	0.0	0.05	0.02	0.02	0.03	0.01
TMRE *	0.01	0.07	0.16	1.0	0.19	0.0	0.0	0.0	0.0	0.0	0.86	0.0	0.01	0.0	0.0	0.0	0.03	0.01	0.0	0.05	0.0
PE-Fire 640	0.0	0.06	0.24	0.19	1.0	0.06	0.07	0.01	0.01	0.0	0.16	0.01	0.11	0.1	0.17	0.14	0.49	0.24	0.0	0.1	0.1
APC-Fire 750	0.0	0.0	0.0	0.0	0.06	1.0	0.18	0.28	0.0	0.0	0.0	0.01	0.78	0.04	0.16	0.18	0.11	0.19	0.0	0.37	0.37
Brilliant Ultraviolet 737	0.0	0.01	0.04	0.0	0.07	0.18	1.0	0.32	0.05	0.02	0.01	0.01	0.12	0.16	0.31	0.18	0.1	0.14	0.01	0.11	0.32
Brilliant Ultraviolet 805	0.01	0.02	0.01	0.0	0.01	0.28	0.32	1.0	0.14	0.04	0.01	0.02	0.28	0.05	0.05	0.02	0.02	0.03	0.01	0.13	0.04
Brilliant Ultraviolet 496	0.13	0.23	0.1	0.0	0.01	0.0	0.05	0.14	1.0	0.1	0.03	0.43	0.03	0.01	0.02	0.0	0.01	0.01	0.22	0.01	0.0
Brilliant Violet 421	0.01	0.03	0.01	0.0	0.0	0.0	0.02	0.04	0.1	1.0	0.0	0.25	0.02	0.0	0.17	0.0	0.0	0.0	0.69	0.0	0.0
PE	0.03	0.25	0.47	0.86	0.16	0.0	0.01	0.01	0.03	0.0	1.0	0.03	0.01	0.01	0.01	0.0	0.03	0.01	0.01	0.06	0.0
Brilliant Violet 480	0.05	0.16	0.05	0.0	0.01	0.01	0.01	0.02	0.43	0.25	0.03	1.0	0.03	0.0	0.05	0.0	0.01	0.01	0.67	0.01	0.0
APC-Fire 810	0.0	0.01	0.02	0.01	0.11	0.78	0.12	0.28	0.03	0.02	0.01	0.03	1.0	0.03	0.15	0.16	0.21	0.21	0.03	0.25	0.14
RealBlue 744	0.03	0.04	0.06	0.0	0.1	0.04	0.16	0.05	0.01	0.0	0.01	0.0	0.03	1.0	0.05	0.02	0.01	0.02	0.0	0.34	0.19
Brilliant Violet 711	0.0	0.01	0.04	0.0	0.17	0.16	0.31	0.05	0.02	0.17	0.01	0.05	0.15	0.05	1.0	0.24	0.18	0.24	0.12	0.13	0.17
Alexa Fluor 700 *	0.0	0.0	0.0	0.0	0.14	0.18	0.18	0.02	0.0	0.0	0.0	0.0	0.16	0.02	0.24	1.0	0.34	0.68	0.0	0.05	0.67
APC	0.0	0.01	0.05	0.03	0.49	0.11	0.1	0.02	0.01	0.0	0.03	0.01	0.21	0.01	0.18	0.34	1.0	0.65	0.0	0.04	0.22
Spark NIR 685	0.0	0.0	0.02	0.01	0.24	0.19	0.14	0.03	0.01	0.0	0.01	0.01	0.21	0.02	0.24	0.68	0.65	1.0	0.01	0.05	0.38
Pacific Blue	0.02	0.07	0.02	0.0	0.0	0.0	0.01	0.01	0.22	0.69	0.01	0.67	0.03	0.0	0.12	0.0	0.0	0.01	1.0	0.0	0.0
PE-Cy7	0.0	0.02	0.03	0.05	0.1	0.37	0.11	0.13	0.01	0.0	0.06	0.01	0.25	0.34	0.13	0.05	0.04	0.05	0.0	1.0	0.15
Zombie NIR	0.0	0.0	0.01	0.0	0.1	0.37	0.32	0.04	0.0	0.0	0.0	0.0	0.14	0.19	0.17	0.67	0.22	0.38	0.0	0.15	1.0

C

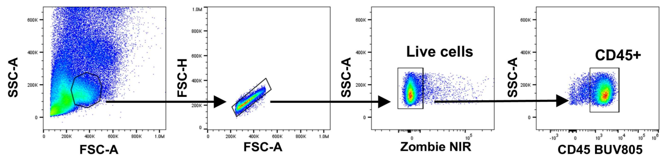

Figure S4. Validation of spectral co-resolvability in immunometabolic profiling panels, related to Figure 4 and STAR Methods.

(A) Representative flow cytometry plots demonstrating pairwise resolution of co-stained metabolic probes. **(B)** Comprehensive FluoroFinder computational matrix quantifying spectral similarity among 4 metabolic probes, 16 surface markers, and 1 viability dye. **(C)** Gating strategy.

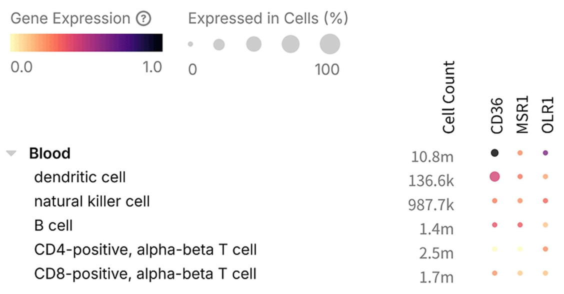

Figure S5. scRNA-seq analysis of lipid-uptake receptor expression across human peripheral blood immune subsets, related to Figure 5 and 6.

Analysis of *CD36*, *MSR1* (CD204), and *OLR1* (LOX-1) expression across functionally defined immune cell subsets using published human peripheral blood mononuclear cell (PBMC) scRNA-seq data (CZI Cellxgene platform: cellxgene.cziscience.com).

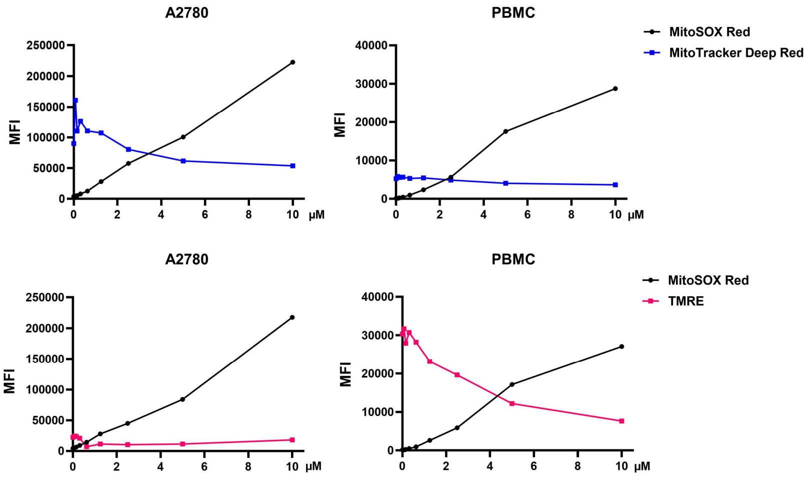

Figure S6. Effect of MitoSOX Red staining concentration on mitochondrial membrane potential, related to Figure 3 and 4.

Dose titration experiments of MitoSOX Red to assess its effect on TMRE and MitoTracker Deep Red, conducted in both A2780 cells and PBMCs.

Table S1 Antibody panel for metabolic co-staining

Specificity	Fluorochrome	Purpose	Clone	Manufacturer	Catalog NO.	Dilution of Stock
CD3	PE-Fire640	Pan T cell, NKT-Like cells	SK7	Biolegend	344859	1:100
CD8	APC-Fire750	CD8 T, NK, and NKT-Like cells	SK1	Biolegend	344746	1:100
CD4	Brilliant Ultraviolet 737	CD4 T and NKT-Like cells	SK3	BD Biosciences	612749	1:100
CD45	Brilliant Ultraviolet 805	Leukocytes	HI30	BD Biosciences	612892	1:100
CD56	Brilliant Ultraviolet 496	Pan NK cell, γδ T cell activation	NCAM16.2	BD Biosciences	750479	1:100
CD16	Brilliant Violet 421	Monocyte, NK cell, and dendritic cell differentiation	B73.1	Biolegend	630723	1:100
NKG2C	PE	NK cell differentiation	S19005E	Biolegend	375003	1:100
IgD	Brilliant Violet 480	B cell differentiation	IA6-2	BD Biosciences	566187	1:400
CD27	APC-Fire810	T and B cell differentiation	0323	Biolegend	302863	1:50
CD19	RealBlue 744	B cells	SJ25C1	BD Biosciences	570469	1:100
CCR7	Brilliant Violet 711	T cell differentiation	G043H7	Biolegend	353227	1:50
CD45RA	Alexa Flour 700	T cell and dendritic cell differentiation	HI100	Biolegend	304119	1:25
CD25	APC	Regulatory T cells	BC96	Biolegend	302609	1:50
CD127	Spark NIR685	Cytokine receptor; T cell differentiation	A109D5	Biolegend	351367	1:100
CD57	Pacific Blue	NK and CD8+ T cell immune senescence	HNK-1	Biolegend	359607	1:200
CD28	PE-Cy7	T cell and NK cell differentiation	CD28.2	Biolegend	302926	1:50
Viability	Zombie NIR	Viability		Biolegend	423105	1:200
