# Supplementary material for: Application of spectral flow cytometry for comprehensive detection of immune metabolism in patient-derived microsamples
Source: Cell Rep Methods. 2026 Mar 16;6(3):101330. doi: 10.1016/j.crmeth.2026.101330 (PMC13030956; doi:10.1016/j.crmeth.2026.101330)
Supplement: Document S2. Article plus supplemental information [file mmc2.pdf]

Cell Reports Methods

Article

Application of spectral flow cytometry for comprehensive detection of immune metabolism in patient-derived microsamples

Graphical abstract

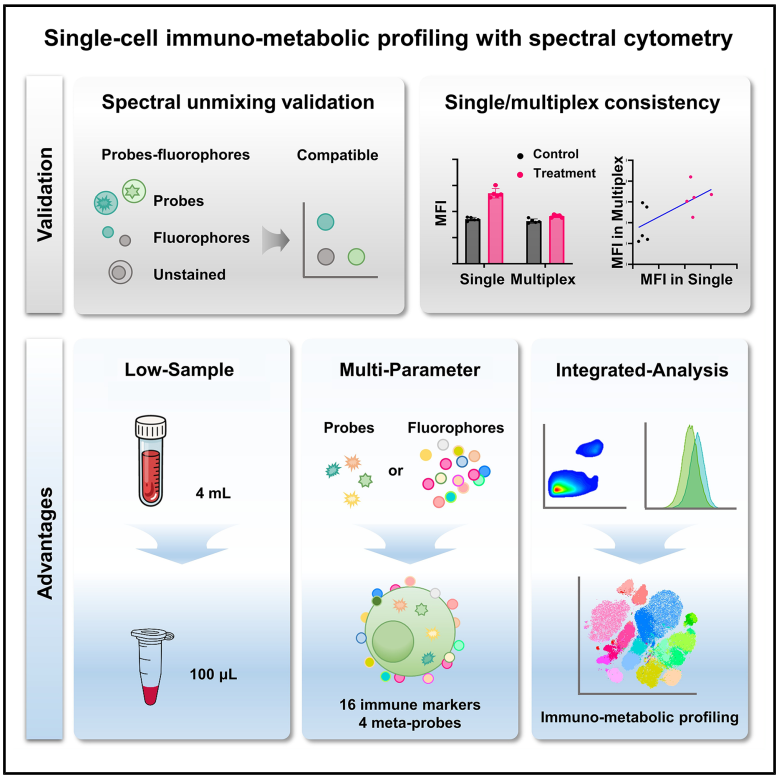

Authors

Yang Bai, Yuqing Wang, Yicheng Fu, ..., Huilin Liu, Xianlong Li, Jie Zhang

Correspondence

liuhuilin202110@163.com (H.L.), xianlong_li@bjmu.edu.cn (X.L.), zhangjie90905138@bjmu.edu.cn (J.Z.)

In brief

Bai et al. develop a spectral flow cytometry platform for high-dimensional analysis of single-cell immunometabolic signatures using small amounts of peripheral blood. Application to samples from patients with heart failure suggests a metabolic shift toward glucose use in T cell subsets, demonstrating the method's potential for precision studies of immunometabolism in disease.

Highlights

- A spectral flow cytometry platform for single-cell immunometabolic profiling
- The platform enables 20-parameter analysis from 100 μL peripheral blood
- We identify metabolic shifts in lymphocytes from patients with heart failure

Bai et al., 2026, Cell Reports Methods *6*, 101330
March 23, 2026 © 2026 The Author(s). Published by Elsevier Inc.
https://doi.org/10.1016/j.crmeth.2026.101330

CellPress

Cell Reports Methods

CellPress
OPEN ACCESS

Article

Application of spectral flow cytometry for comprehensive detection of immune metabolism in patient-derived microsamples

Yang Bai,[1,2,3,4,7] Yuqing Wang,[1,2,3,4,7] Yicheng Fu,[1,7] Zhengyang Guo,[2,3,4] Zhaoyuan Liang,[2,3,4] Liu Yang,[2,3,4] Jiawei Ribaudo,[6] Dan Liu,[2,3,4] Yanfang Li,[2,3,4] Ting Zhang,[2,5] Lixiang Xue,[2,3,4,5] Jianling Yang,[1] Huilin Liu,[1,*] Xianlong Li,[2,3,4,*] and Jie Zhang[2,3,4,5,8,*]

[1]Department of Geriatrics, Peking University Third Hospital, Beijing, China

[2]Center of Basic Medical Research, Institute of Medical Innovation and Research, Peking University Third Hospital, Beijing, China

[3]Cancer Center of Peking University Third Hospital, Beijing, China

[4]Beijing Key Laboratory of Interdisciplinary Research in Gastrointestinal Oncology (BLGO), Peking University Third Hospital, Beijing, China

[5]Biobank, Peking University Third Hospital, Beijing, China

[6]Biologic and Materials Sciences & Prosthodontics, University of Michigan School of Dentistry, Ann Arbor, MI, USA

[7]These authors contributed equally

[8]Lead contact

*Correspondence: liuhuilin202110@163.com (H.L.), xianlong_li@bjmu.edu.cn (X.L.), zhangjie90905138@bjmu.edu.cn (J.Z.)

https://doi.org/10.1016/j.crmeth.2026.101330

MOTIVATION Immunometabolism underpins a wide range of disease states. Spectral flow cytometry has advanced immunophenotyping, but its potential for assaying single-cell, multi-parametric metabolic characteristics has not been fully unlocked. Here, we leverage the multiplexing capacity of spectral flow cytometry to establish a platform for simultaneous assessment of multiple metabolic and immune features with limited sample consumption.

SUMMARY

Single-cell metabolic characteristics are powerful indicators of cellular physiological and pathological states. Flow cytometry enables high-throughput single-cell metabolic profiling and immunophenotyping; however, spectral overlap impedes simultaneous detection of multi-parametric features. This limitation imposes larger sample requirements and increased variabilities due to metabolic dynamics—constraints acutely magnified in studies utilizing clinical microsamples. To overcome these shortcomings, we developed a spectral flow cytometry platform for immunometabolic features, using 13 dual-probe combinations and 11 fluorophore probes. Requiring only 100 μL of whole blood per assay, this platform enables concurrent detection of 4 metabolic biomarkers and 16 immune markers. Application to patients with heart failure revealed heterogeneous metabolic landscapes across 20 immune subpopulations, showing reduced frequencies of naive T cells and NK-like T cells, with a metabolic shift from fatty acid dependence to glucose avidity. Our framework captures metabolic interactions previously inaccessible by sequential detection and will help to enable precision immunometabolism research.

INTRODUCTION

Cellular metabolism plays a pivotal role in physiological and pathological processes, with dysregulation associated with diabetes, cancer, and inflammation.[1–4] Metabolic networks function through tightly coordinated pathways: glucose uptake fuels glycolysis, fatty acid oxidation supports mitochondrial respiration, and redox balance regulates various signaling cascades.[1] Metabolic features of distinct cell types provide critical insights into their functional states and dynamic reprogramming across physiological and pathological conditions.[5–7]

Conventional metabolic profiling relies mainly on three techniques. The Seahorse Analyzer measures the extracellular acidification rate (ECAR) and oxygen consumption rate (OCR) using metabolic inhibitors (e.g., 2-deoxy-D-glucose and oligomycin A).[8] Mass spectrometry (MS)-based metabolomics quantifies metabolite concentrations[9];while chromogenic substrate assays monitor metabolic enzyme activity.[10] However, these methods

© 2026 The Author(s). Published by Elsevier Inc.
This is an open access article under the CC BY license (http://creativecommons.org/licenses/by/4.0/).

Table 1. Characteristics of flow cytometry-compatible metabolic probes

Fluorescent dye	Purpose	Ex/Em (nm)	Working concentration
BODIPY C12	fatty acid uptake	508/514	1 μM
BODIPY C16	fatty acid uptake	505/512	0.2 μM
MitoTracker Green	mitochondrial mass	490/516	10 nM
H2-DCFDA	intracellular ROS	492–495 /517–527	100 nM
NBD-Cholesterol (NBD)	cholesterol uptake	469/537	10 μM
2-NBDG	glucose uptake	465/540	100 μM
pHrodo Red	pH indicator	560/587	50 nM
DHE	superoxide anion	518/605	1 μM
MitoSox Red	mitochondrial ROS	396/610	3 μM
TMRE	mitochondrial membrane potential (MMP)	549/574	5 nM
MitoTracker Deep Red	mitochondrial mass and MMP	633/665	10 nM

typically require large numbers of cells and lack single-cell resolution. Flow cytometry, a high-throughput, single-cell analysis platform, overcomes this limitation by enabling detection of mitochondrial mass (MitoTracker), mitochondrial membrane potential (tetramethylrhodamine, ethyl ester, TMRE), reactive oxygen species (ROS: MitoSOX/DHE), metabolite uptake (glucose uptake: 2-NBDG; lipid metabolism: BODIPY), and intracellular pH (pHrodo), using various specific metabolic probes (Table 1).[11–14] Combined with surface marker staining, flow cytometry can characterize metabolic features within specific immune cell subsets without prior sorting.

Conventional flow cytometry is fundamentally limited by the spectral overlap when attempting simultaneous multi-parametric metabolic profiling (Table 1). This imposes four major constraints: (1) increased sample consumption, requiring separate aliquots for each probe; (2) time-consuming processing prone to variations due to rapid metabolic dynamics; (3) challenges when co-staining with surface markers; and (4) difficulty in correlating different metabolic parameters. These limitations are evident in precious clinical microsamples.

To address this gap, we leverage the multiplexing capacity of spectral flow cytometry to achieve simultaneous spectral resolution among diverse metabolic probes and between metabolic probes and traditional fluorophores. While spectral flow cytometry has revolutionized high-dimensional immunophenotyping through superior fluorescence resolution,[15–17] its potential for single-cell multi-metabolic profiling remains largely untapped. Here, we established a systematic pipeline for assessing spectral compatibility between probe-probe and probe-fluorophore pairs, identifying 13 dual-probe combinations and 11 co-detection panels with traditional fluorophores, all enabling at least 2 metabolic parameters to be quantified in a single tube. This platform was successfully deployed to: (1) resolve drug/genetic perturbation-induced mitochondrial dysfunction (via TMRE-based mitochondrial membrane potential quantification) and ROS dynamics (H2-DCFDA/MitoSOX); and (2) contrast immune-metabolic reprogramming in heart failure (HF) microenvironments. Notably, this approach reduces sample consumption by 75%, while enabling comprehensive single-cell metabolic profiling in low-cellularity clinical specimens.

Moreover, by integrating multiplexed metabolic probing with immunophenotyping, we establish a novel analytical framework that categorizes immune cell subsets through their metabolic signatures. Critically, this integrated approach not only enables systematic interrogation of how metabolic rewiring governs immune cell behavior in disease pathogenesis but also provides a systems-level perspective on immunometabolic regulation.

RESULTS

Spectral flow cytometry-based validation platform for metabolic probe compatibility

To establish a robust platform for multiplex metabolic detection by spectral flow cytometry, we first evaluated eleven commercially available probes selected for spectral diversity: six 488 nm-excited probes (BODIPY C12, BODIPY C16, MitoTracker Green, H2-DCFDA, NBD-cholesterol, and 2-NBDG; 510–540 nm emission), four 561 nm-excited probes (pHrodo Red, DHE, MitoSOX Red, and TMRE; 575–605 nm emission), and one 633 nm-excited probe (MitoTracker Deep Red; 665 nm emission). All probes require live-cell labeling. Reference spectra were established using individually stained A2780 tumor cells on the ID7000 spectral cytometer (Figure S1).

Before assessing multiplex compatibility, we systematically optimized key staining variables to ensure reproducible single-probe signals. A critical parameter for consistent staining is the probe-to-cell ratio. Titration experiments confirmed that the mean fluorescence intensity (MFI) increased with probe concentration at a constant cell number (Figures S2A and S2B). Notably, under identical staining conditions, cell number significantly influenced the MFI, with marked differences between samples containing 10^5 cells versus those with 10^6 cells (Figure S2C). Furthermore, cell size also influences the staining efficiency. Therefore, it is necessary to determine the optimal probe concentration for distinct cell types or activation states to ensure reproducible staining outcomes.

To address practical constraints in multiparametric workflows, we evaluated the effects of short-term storage and fixation. Stained cells stored at 4°C in the dark for 3 h maintained MFI within ±25% for all 11 probes (Figure S2D), supporting that short-term storage does not compromise overall reliability.

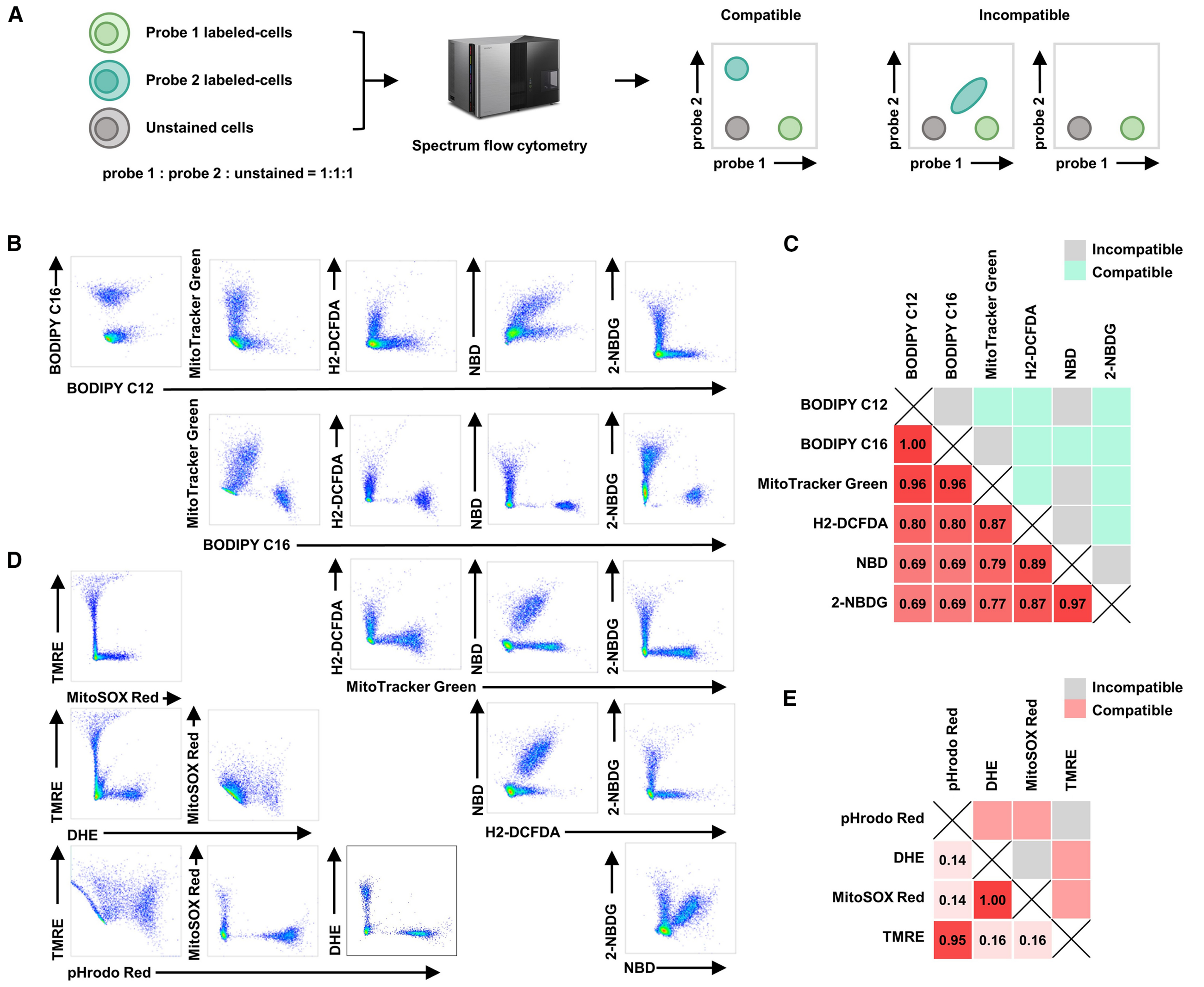

Figure 1. Assessing spectral resolvability of metabolic probes for multiparametric cytometry

(A) Experimental workflow for evaluating probe co-detection.

(B and D) Representative density plots demonstrating spectral overlap for pairwise probe combinations within the (B) FITC and (D) PE channel.

(C and E) Complementary validation matrices showing computational spectral similarity scores from FluoroFinder (lower left quadrant) versus empirical resolvability determined by unmixing (upper right quadrant). Gray indicates non-resolvable pairs; colored tiles indicate resolvable pairs (C, green; E, red).

In contrast, fixation with paraformaldehyde markedly reduced signals from viability-dependent probes such as MitoTracker Green, TMRE, and H2-DCFDA (Figure S2E), underscoring the necessity of live-cell analysis for accurate metabolic readouts.

Having established standardized staining conditions, we next developed an experimental strategy to empirically evaluate spectral overlap between probes—a critical step for reliable multiplexing. A key challenge lies in the holistic labeling of probes, which makes it difficult to clearly distinguish between the negative and positive cell populations. To address this, we adopted a dual experimental strategy mixing probe 1-stained, probe 2-stained, and unstained cells (1:1:1 ratio), using unstained cells to define negative gates. Probe compatibility was determined by orthogonal signal distribution in bivariate plots, where distinct clusters along both axes indicated resolvable pairs, while overlapping clusters denoted incompatibility (Figure 1A).

This empirical analysis revealed significant discrepancies from computational predictions. In the FITC channel (488 nm), we identified 9 compatible and 6 incompatible probe pairs (Figures 1B and 1C), contrasting FluoroFinder algorithmic outputs. For PE-channel probes (561 nm), four compatible pairs (pHrodo Red/DHE, pHrodo Red/MitoSOX Red, DHE/TMRE, and MitoSOX Red/TMRE) and two incompatible pairs (pHrodo Red/TMRE and DHE/MitoSOX Red) were validated (Figures 1D and 1E). These findings emphasize the necessity of empirical validation over purely algorithmic predictions for probe compatibility assessment.

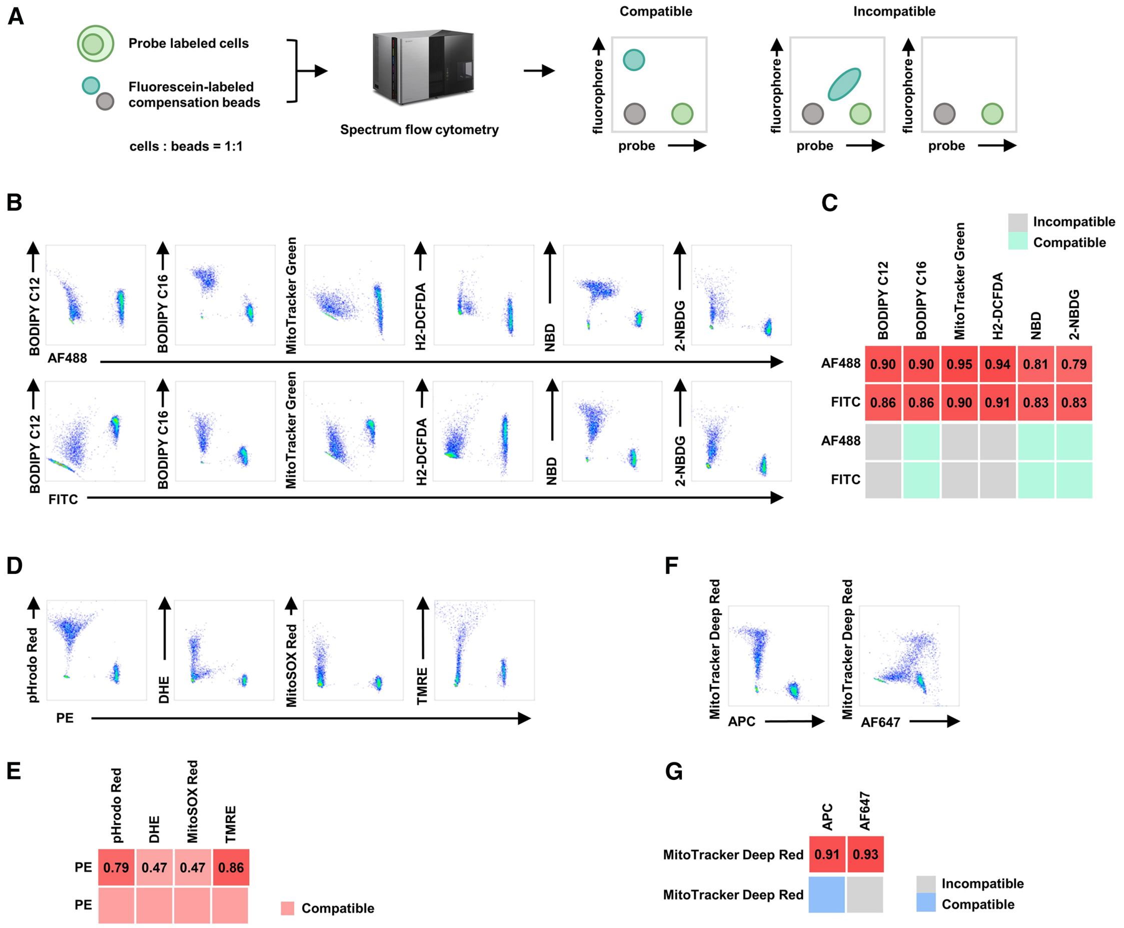

Figure 2. Validation framework for spectral co-resolvability of metabolic probes and fluorophore conjugates

(A) Experimental workflow for co-detection assessment.

(B, D, and F) Representative flow cytometry density plots demonstrating pairwise mixing between metabolic probes and fluorophores in the (B) FITC/AF488, (D) PE, and (F) APC/AF647 channel.

(C, E, and G) Validation matrices per channel: upper, computational spectral similarity by FluoroFinder; lower, empirically determined resolvability. Gray tiles indicate non-resolvable pairs; colored tiles confirm resolvable pairs. (C) FITC/AF488, green; (E) PE, red; and (G) APC/AF647, blue.

Establishment of a systematic framework for evaluating metabolic probe compatibility with conventional fluorophores in immunometabolic profiling

Simultaneous detection of immune subset identity and metabolic states requires co-staining with surface markers and functional probes. We evaluated spectral compatibility between metabolic probes and conventional fluorophores across three key flow cytometry channels—488-nm excitation channel (commonly FL1 for FITC/AF488 detection), 561-nm excitation channel (commonly FL2 for PE detection), and 633-nm excitation channel (commonly FL4 for APC/AF647 detection). Using a hybrid validation system combining fluorescent microspheres (stained with surface marker fluorophores) and probe-labeled cells, we identified resolvable probe-fluorophore pairs based on orthogonal signal distribution in bivariate plots (Figure 2A).

In the 488-nm channel (FL1), empirical testing revealed three metabolically informative probes compatible with FITC/AF488 fluorophores: BODIPY C16, 2-NBDG, and NBD-cholesterol (NBD). In contrast, BODIPY C12, MitoTracker Green, and H2-DCFDA exhibited prohibitive spectral overlap, precluding their concurrent use (Figures 2B and 2C). Within the 561-nm channel (FL2), pHrodo Red, DHE, MitoSOX Red, and TMRE demonstrated robust compatibility with PE fluorophores (Figures 2D and 2E). For the 633-nm channel (FL4), MitoTracker Deep Red was compatible with APC but incompatible with AF647, underscoring dye-specific resolution limits (Figures 2F and 2G). Notably, the experimental results diverged from FluoroFinder algorithmic predictions for about one-third of probe-fluorophore pairs, highlighting the necessity of empirical validation despite computational guidance.

Simultaneous assessments of mitochondrial function and oxidative stress

Mitochondrial function and oxidative stress represent fundamental determinants of cellular health, serving as sensitive indicators of metabolic activity, redox homeostasis, and apoptotic susceptibility. To enable simultaneous assessment of these critical

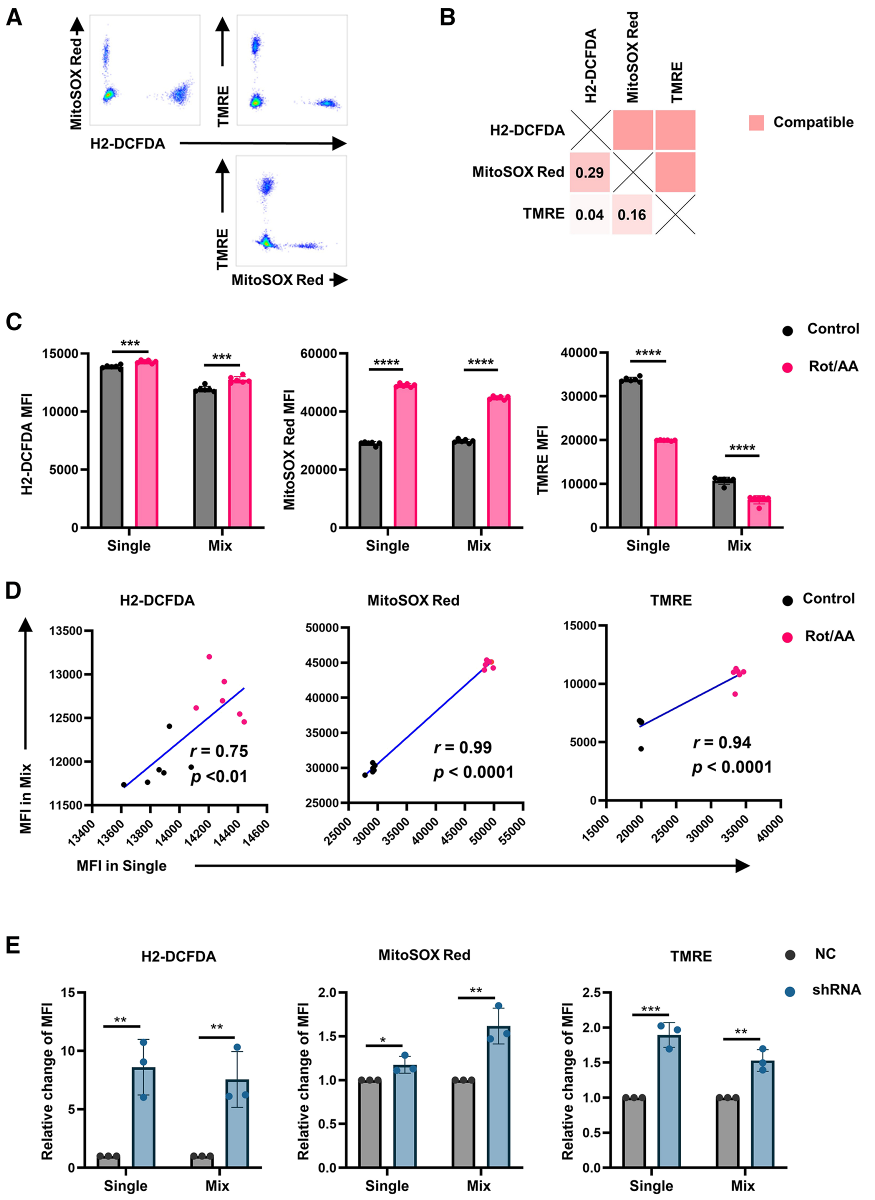

Figure 3. Validation of three metabolic probes for simultaneous assessment of mitochondrial activity and oxidative stress

(A) Representative flow cytometry density plots demonstrating spectral resolution of co-stained probes.

(B) Validation matrix: lower left quadrant, computational spectral similarity by FluoroFinder; upper right quadrant, empirical resolvability determination (red: resolvable pairs).

(C) Mean fluorescence intensity (MFI; mean ± SD) of individual probes in control versus rotenone/antimycin A (Rot/AA)-treated groups (n = 6).

(D) Correlation analysis of MFI between single-stain and multiplexed conditions across probes (Spearman's r).

(E) MFI of probes (mean ± SD) in control versus EZH2-knockdown (EZH2-sh) groups under single and multiplexed staining. *, $p < 0.05$; **, $p < 0.01$; ***, $p < 0.001$; ****, $p < 0.0001$.

parameters, we established a quantitative platform employing four complementary fluorescent probes targeting distinct but interconnected aspects of redox biology: H2-DCFDA for cytosolic ROS, MitoSOX Red for mitochondrial superoxide, TMRE for mitochondrial membrane potential, and MitoTracker Deep Red for mitochondrial mass. These probes collectively provide a multifaceted evaluation of cellular oxidative stress and mitochondrial integrity.

Initial spectral validation confirmed the compatibility of MitoSOX Red and TMRE within the PE channel (Figures 1D and 1E). Further integration with H2-DCFDA and MitoTracker Deep Red demonstrated pairwise spectral separability (Figures S3A and S3B). However, functional validation using rotenone and antimycin A—pharmacological agents that disrupt mitochondrial complexes I/III and induce ROS overproduction[18]—revealed discordant results for MitoTracker Deep Red. Although the single-stain controls showed increased mitochondrial mass upon treatment, multiplexed measurements paradoxically indicated a decrease (Figures S3C and S3D). This inconsistency prompted the exclusion of MitoTracker Deep Red, yielding an optimized three-probe panel (H2-DCFDA, MitoSOX Red, and TMRE) with preserved spectral resolution and functional reliability (Figures 3A and 3B).

In rotenone/antimycin A-treated A2780 cells, the optimized panel detected elevated ROS levels and reduced mitochondrial membrane potential, consistent with mitochondrial dysfunction (Figure 3C). Furthermore, to rigorously evaluate probe performance under different experimental conditions, we conducted systematic comparisons of MFI values between the single-stained and co-stained samples. Multiplexed measurements strongly correlated with single-stain controls (H2-DCFDA: r = 0.75; MitoSOX Red: r = 0.99; TMRE: r = 0.94; Figure 3D), confirming technical robustness.

To further validate the broad applicability of our multiparametric probe system, we employed complementary experimental approaches beyond pharmacological modulation. Specifically, we employed an EZH2-knockdown MDA-MB-231 breast cancer model. shRNA-mediated EZH2 depletion induced ROS elevation and mitochondrial membrane potential hyperpolarization (Figure 3E), aligning with reported EZH2-mitochondria crosstalk.[19] Crucially, multiplexed detection replicated single-stain trends (Figure 3E), underscoring the panel's reliability across pharmacological and genetic models.

Collectively, these results demonstrate the utility of a three-probe panel (H2-DCFDA, MitoSOX Red, and TMRE) for

multiplexed evaluation of oxidative stress and mitochondrial function. The strategy reliably captured redox perturbations in diverse experimental systems, suggesting its applicability for mechanistic studies of mitochondrial pathophysiology.

Metabolic profiling with immune subtype analysis in peripheral blood mononuclear cells

To enable single-cell metabolic characterization of immune subpopulations, we combined four functional probes assessing glucose uptake (2-NBDG), fatty acid uptake (BODIPY C12), mitochondrial ROS (MitoSOX Red), and mitochondrial membrane potential (TMRE). These were selected based on their spectral compatibility and biological relevance. Single-stain controls confirmed distinct spectral separation between all probe pairs (Figure S4A). Subsequent 16 surface markers (CD3, CD4, CD8, CD19, CD56, etc.) demonstrated unimpaired signal discrimination between metabolic probes and immunophenotypic fluorophores, enabling simultaneous detection of metabolic states and immune identity (Figure S4B). To match antibody fluorescence intensities under unified voltages, a key optimization was empirically reducing the probe concentrations (e.g., TMRE to 5 nm, 400-fold below typical concentrations). Supporting this, our data demonstrate that metabolic probe MFI increases with concentration (Figure S2A).

Multiparametric analysis resolved peripheral blood mononuclear cells (PBMCs) into 20 functionally distinct subsets: 4 NK cell clusters (Pop 1–4), 2 NK-like T cell clusters (Pop 5–6), 1 innate lymphoid cell (ILC) cluster (Pop7), 5 $CD8^+$ T cell clusters (Pop 8–12), 4 $CD4^+$ T cell clusters (Pop 13–16), 2 B cell clusters (Pop 17–18), and 2 undefined clusters (Pop 19–20; Figures 4A and 4B). Validation experiments using mixed-stain specimens confirmed a strong correlation (Pearson's $r > 0.99$) between metabolic probe MFI in single-parameter versus multiplexed assays across all subsets (Figures 4C and 4D), verifying the panel's capacity to faithfully capture metabolic heterogeneity at single-cell resolution.

Heterogeneous energetic landscapes across immune subpopulations

To elucidate the interplay between immunophenotypic profiles and metabolic heterogeneity, we conducted a comparative analysis of metabolic features across distinct immune cell subsets. We found broad comparability in glucose and lipid uptake across most cell populations (Figures 5A and 5B). Glucose uptake predominated in NK cells, while fatty acid uptake showed minimal inter-lineage variation except for elevated levels in Pop19 and 20 (Figures 5A and 5B). Notably, both senescent $CD8^+$ and $CD4^+$ T cells (Pop12 and 16) displayed progressive fatty acid dependency, while glucose uptake followed a biphasic pattern peaking at intermediate activation states (Figures 5A and 5B). Specialized populations demonstrated extreme substrate polarization, with Pop8 favoring glucose versus Pop20's lipid preference (Figures 5A and 5B). Mitochondrial assessment revealed cell-type specific functional signatures: T cells maintained the highest oxidative stress (Figure 5C), while B cells exhibited superior membrane potential (Figure 5D). Parallel trends in mitochondrial ROS and membrane potential across subsets suggested coupled regulation of oxidative metabolism and bioenergetic capacity.

To enable comprehensive metabolic phenotyping, unsupervised clustering integrated data from four metabolic probes, resolving immune cells into four distinct subpopulations: glucose-avid, ROS-high activated oxidative phenotype (cluster 1), lipid-specialized mitochondria-depressed subsets (cluster 2), dual-substrate plastic populations (cluster 3), and nutrient-deprived with mitochondrial dysfunction subsets (cluster 4, Figures 5E and 5F). While 90% of the cells exhibited polarized distribution between clusters 1 and 3, rare disease-associated niches emerged: cluster 2 dominated lipid-dysregulated Pop20, whereas cluster 4 accumulated in senescent T cells (Figure 5G). Subpopulation-specific stratification uncovered distinct metabolic flexibility in NK/T cells (balanced clusters 1/3), coordinated substrate utilization in naive B/NK-like T cells (70% cluster 3), and glucose-driven effector priming during B cell maturation (Pop17→18; Figure 5G).

This high-resolution mapping reveals conserved metabolic circuits across immune subsets, establishing a quantitative framework for immunometabolic analysis.

HF drives lymphocyte metabolic polarization, linking terminal differentiation to metabolic inflexibility and mitochondrial stress

While HF is known to induce systemic inflammation and immune dysregulation,[20] the metabolic adaptations of lymphocyte subsets involved remain poorly characterized. Using our validated 21-parameter spectral flow cytometry panel on cryopreserved PBMCs from patients with HF and matched controls, we first identified and confirmed the overall frequencies of the major lymphocyte population involved (Figure 6A). In addition, further immunophenotyping revealed significant subset-specific remodeling in patients with HF. Early differentiated populations suffered marked depletion, with $CD8^+$ naive T cells (Pop8) and NK-like T cells (Pop5-6) declining, respectively, in patients with HF (Figure 6B). Conversely, terminally differentiated $CD57^+$ NK cells (Pop4) expanded, accompanied by non-significant increases in $CD57^+$ $CD8^+$ (Pop12) and $CD57^+CD4^+$ T cells (Pop16, Figure 6B), suggesting systemic accumulation of senescent lymphocytes in HF.

Metabolically, HF induced lineage-specific perturbations despite preserved global glucose uptake (Figure 6C). Fatty acid uptake collapsed in ILCs (Pop7) and Pop20 (Figure 6D), indicating compromised lipid metabolism. Mitochondrial dysfunction emerged as a hallmark of HF immunopathology, with NK-like T cells (Pop5) developing severe oxidative stress (MitoSOX MFI, Figure 6E), while Pop20 exhibiting catastrophic membrane potential loss (TMRE MFI, Figure 6F).

Unsupervised clustering of single-cell metabolic states uncovered two divergent reprogramming trajectories. Conventional T cells and NK-like T cells (Pop5) shifted from fatty acid uptake cluster 3 to glucose-avid cluster 1, adopting a hyperactivated phenotype characterized by elevated mitochondrial ROS despite reduced bioenergetic capacity (Figures 6G and 6I). This metabolic polarization aligns with increased glycolytic flux required for cytotoxic effector functions.[21,22] Conversely, for Pop20, the metabolic phenotype transitioned from cluster 2 to cluster 3, characterized by reduced lipid uptake and elevated mitochondrial membrane potential (Figures 6H and 6I). No significant changes were observed for cluster 4 (Figure 6J).

CellPress
OPEN ACCESS

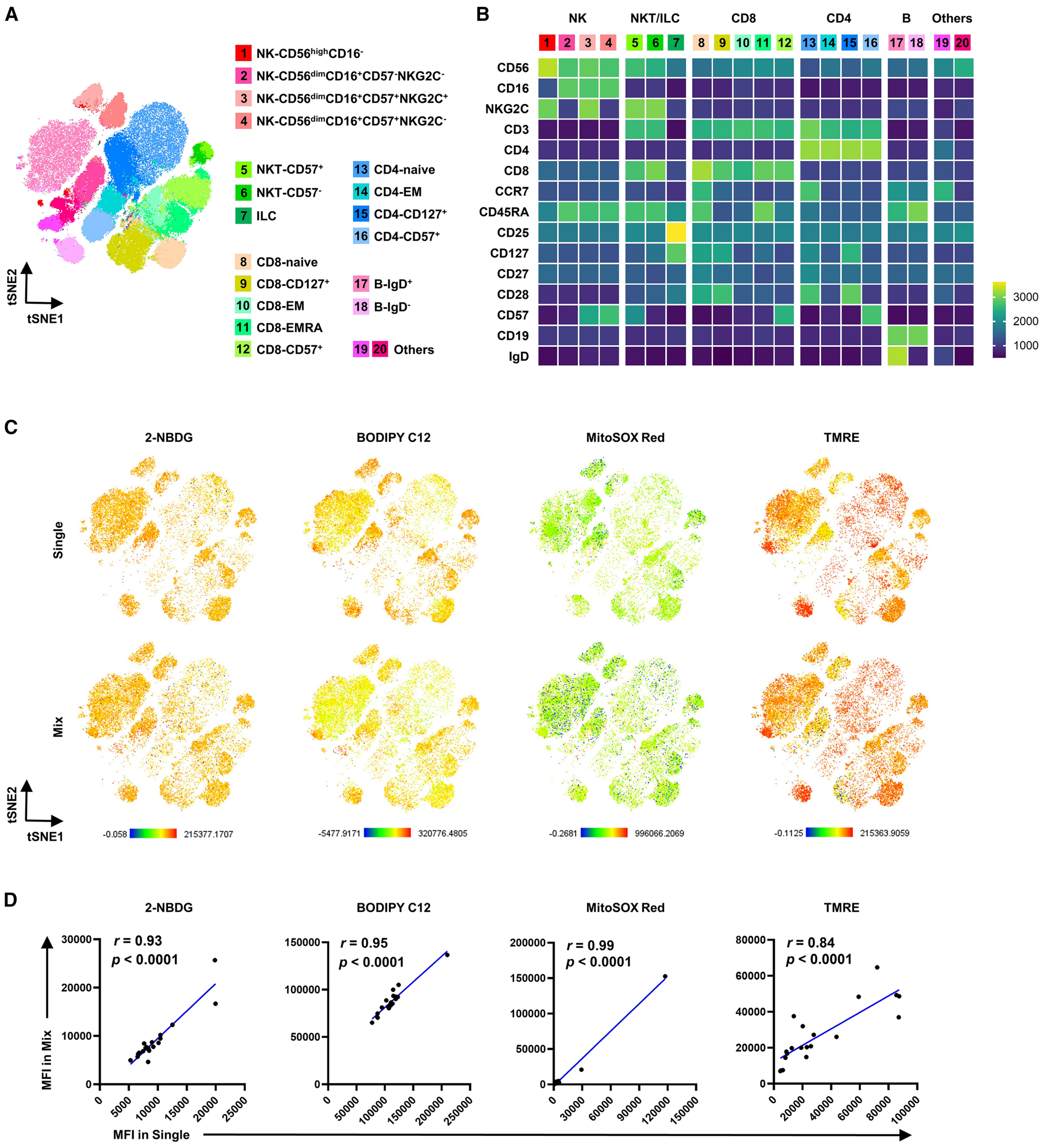

Figure 4. Validation of multiplexed metabolic probe co-staining in immune cell subsets from peripheral blood of healthy donors

(A) Dimensionality reduction (t-SNE) depicting immune cell subset identification.

(B) Expression levels of subset-defining surface markers within corresponding clusters.

(C) Comparative visualization of four metabolic probes (2-NBDG, BODIPY C12, MitoSOX Red, and TMRE) across cell subsets under individual versus multiplexed staining conditions.

(D) Correlation analysis of the MFI for metabolic probes between single-stain and multiplexed conditions across all immune subsets (Spearman's r).

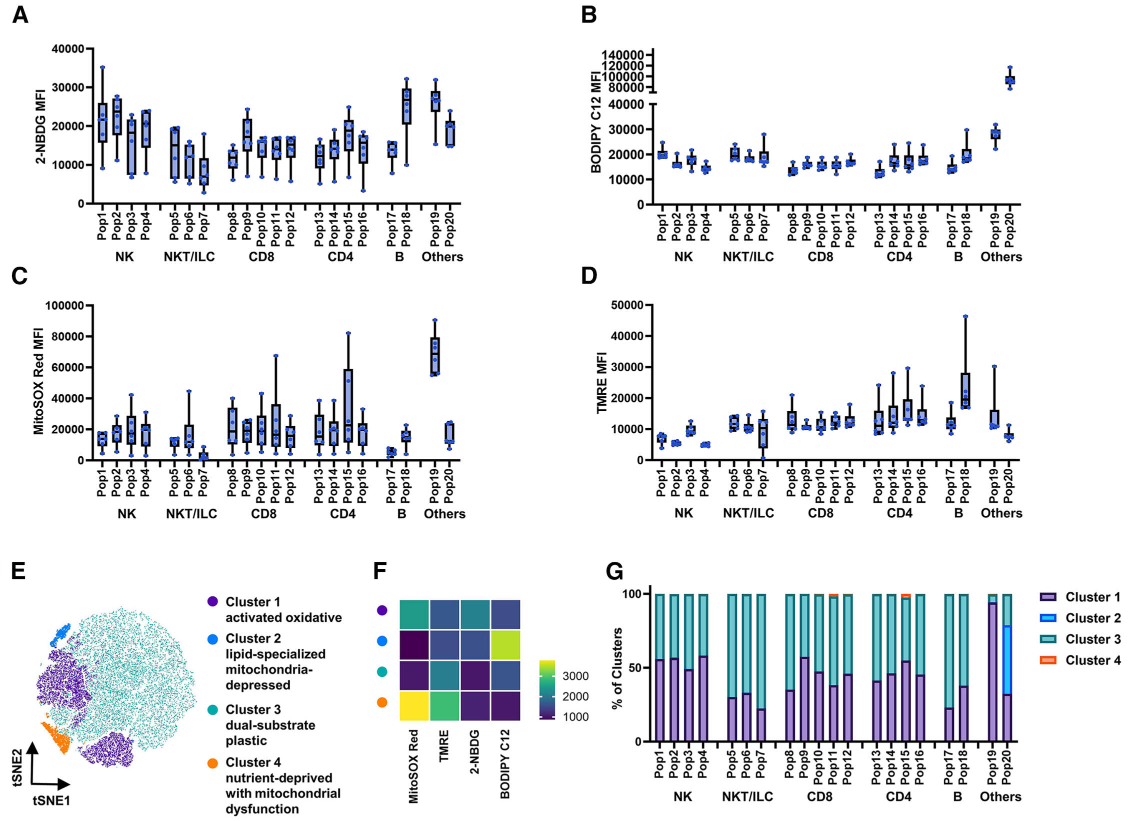

Figure 5. Integrated metabolic feature of immune cell subsets in peripheral blood from healthy donors

(A–F) Analysis of PBMCs from healthy donors (n = 6).

(A–D) MFI of four live-cell metabolic probes across 20 immune cell subsets. (A) glucose uptake (2-NBDG), (B) lipid utilization (BODIPY C12), (C) mitochondrial superoxide (MitoSOX Red), and (D) mitochondrial membrane potential (TMRE).

(E and F) Unsupervised metabolic clustering based on integrated probe signatures (n = 4 clusters) is visualized through (E) dimensionality reduction (t-SNE) and (F) cluster-specific metabolic signature heatmaps.

(G) Proportional distribution of metabolic clusters across all profiled immune subsets.

These findings implicated HF as a key driver for lymphocyte terminal differentiation coupling with metabolic substrate inflexibility and mitochondrial stress. Chronic inflammation may accelerate both metabolic exhaustion and pathological immune activation.

DISCUSSION

Spectral flow cytometry has revolutionized fluorescent antibody/probe panel design by expanding combinatorial diversity, as its ultra-high-resolution spectral unmixing algorithms can distinguish fluorophores with highly similar emission spectra. Yet, challenges in spectral resolution and data analysis persist. Our platform resolves long-standing spectral conflicts, permitting the concurrent detection of glucose/lipid uptake, mitochondrial function, and redox states within a single tube—a capability unattainable with conventional systems. By establishing 12 dual-probe panels, we achieved high-dimensional metabolic profiling with 75% reduced sample requirement. Collectively, this advance establishes a sample-efficient, multi-parametric platform that enables integrative assessment of real-time metabolic interactions, capturing dynamic network crosstalk previously obscured by sequential detection.

We established a framework to resolving computationally overlapping metabolic probe pairs (similarity index > 0.69) via spectral unmixing, enabling resolvable quadplex co-staining. However, co-stain-specific signal drift (MitoTracker Deep Red; Figure S3) highlighted biological artifacts. FMO controls revealed MitoSOX exclusion partially stabilized signals (data not shown), indicating superoxide-dependent oxidation[23] may alter membrane topology independently of spectral interference. While mechanistic details require further investigation, protocol optimization through sequential staining or advanced probes could mitigate this interference. These findings establish two essential validation criteria: (1) technical spectral resolvability via empirical unmixing; and (2) biological consistency between single and multiplexed conditions.

Following co-detection validation, we established a spectral compatibility framework integrating fluorophores and metabolic probes. This assigned key metabolic probes to specific channels: three FITC/AF488-compatible (BODIPY C12, MitoTracker Green, and H2-DCFDA), three PE-compatible (pHrodo, DHE, and MitoSOX Red), and one APC-compatible (MitoTracker Deep Red), enabling simultaneous surface marker and metabolic analysis without sorting. Multiplexed spectral co-staining

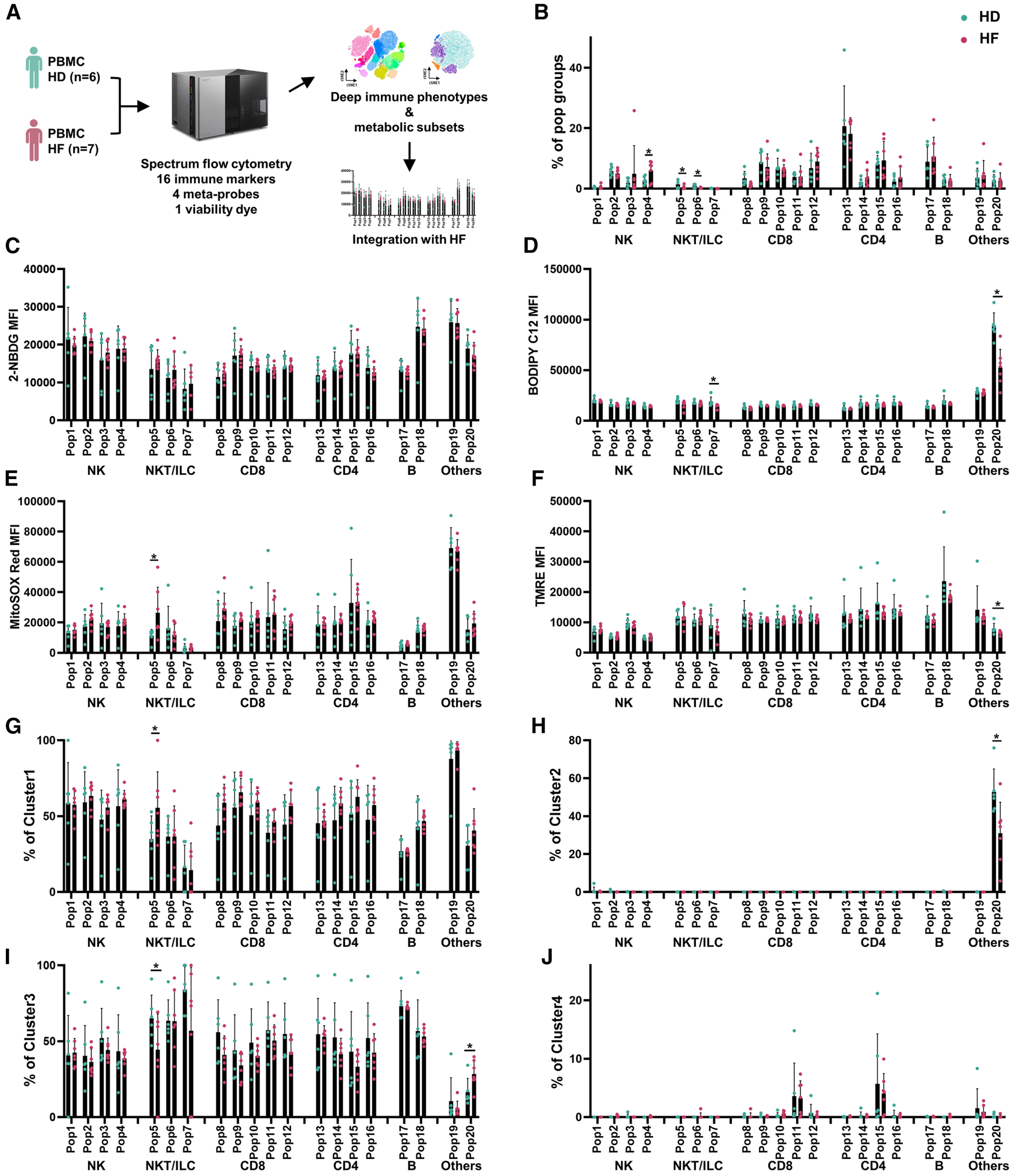

Figure 6. Immunometabolic features of peripheral blood in patients with HF

(A) Experimental workflow for simultaneous immunophenotypic and metabolic analyses.

(B–J) Comparative analysis of peripheral blood from patients with HF (n = 7) versus healthy donors (n = 6); frequencies of 20 immune cell subsets (B); MFI of metabolic probes—glucose uptake (2-NBDG) (C), lipid utilization (BODIPY C12) (D), mitochondrial superoxide (MitoSOX Red) (E), and mitochondrial membrane potential (TMRE) (F); proportional distribution of four integrated metabolic subtypes (clusters 1–4; G–J).

Data represent the mean ± SD; *, $p < 0.05$ (two-tailed t test).

established a novel metabolic subtyping framework, identifying four metabolic clusters (1–4) that map heterogeneity and recapitulate reprogramming events, such as the glycolytic shift (cluster 1 enrichment) accompanying B cell maturation from IgD^{+} naive (Pop17) to IgD^{-} functional (Pop18) states[24,25] and the enrichment of metabolically impaired cluster 4 in senescent T cells.[26,27] Crucially, comprehensive metabolic signatures enabled identity inference for ambiguous populations: Pop20's lipid-utilizing cluster 2 signature and enhanced DC-characteristic scavenger receptor expression (*CD36*/*MSR1*/*OLR1*, Figure S5) strongly suggested a DC lineage,[28] pending confirmation via canonical markers such as CD11c/HLA-DR. Collectively,

our findings reveal metabolic fingerprinting via multiplexed probing as a powerful complementary tool for immune cell classification, offering distinct resolution for phenotypically indistinct or rare populations in clinical contexts.

We successfully applied our platform to analyze the metabolic states of selected immune cells in patients with HF. Our results revealed systemic immunometabolic remodeling: naive subset contraction (e.g., $CD8^+$ T cells) and terminally differentiated effector expansion alongside a metabolic transition from the plastic (cluster 3) to the activated oxidative states (cluster 1, Figure 6), linking immune activation to pro-inflammatory cytokine overproduction.[29] It further connected mTOR inhibitors' cardiac protection to immunomodulation via metabolic reprogramming of T cells.[30–32] While Pop20 exhibited distinct metabolic alterations (Figures 6H and 6I), their pathological significance requires functional validation through phenotyping and targeted pathway interrogation. Nevertheless, the HF paradigm validates our platform's capacity to resolve disease-relevant immunometabolic dysregulation, thereby highlighting the potential for identifying novel therapeutic targets through metabolic-immune modulation.[33]

Our method holds promise for integration with contemporary single-cell metabolic profiling platforms, yet requires strategic optimization to address technical constraints. Critically, while label-free NADH autofluorescence imaging enables quantitative assessment of the cellular metabolic status,[34] its inability to generate reference spectra precludes co-detection with fluorescent metabolic probes (e.g., glycolytic sensors) in spectral cytometry, effectively limiting multiparametric analysis. Antibody-based methods like SCENITH (ATP synthesis profiling)[35] and Met-Flow (metabolic enzyme quantification)[36] can be effectively integrated with our platform by replacing live-cell probes with fixation-compatible alternatives (e.g., MitoTracker Deep Red). However, this modification forfeits the real-time metabolic dynamics captured by our live-cell imaging protocol. A key advantage of our workflow is its ability to functionally stratify metabolically distinct subpopulations for downstream multi-omics analysis. Sorted cells can undergo concurrent single-cell RNA sequencing (scRNA-seq)[37] and viability-preserving metabolomics (e.g., DCFHDA-coupled scMetabolomics),[38] creating multilayered molecular maps. This integrative framework may overcome the reductionist limitations of isolated metabolic parameter measurements by enabling systematic deconvolution of metabolic heterogeneity across biological systems.

In conclusion, we established an integrated spectral cytometry framework that enables simultaneous, multiplexed live-cell metabolic and immunophenotypic profiling. By resolving spectral conflicts and biological artifacts, it uncovers disease-relevant immunometabolic circuits and reveals the potential of metabolic fingerprints as complementary classifiers for immune identity, advancing precision immunometabolism.

Limitations of the study

Here, we established a spectral flow cytometry-based framework for multi-probe metabolic profiling, enabling the analysis of metabolic alterations in limited clinical specimens. While this approach demonstrates significant utility, several limitations warrant consideration. First, although our analysis defined metabolic characteristics across 20 immune subsets using 4 metabolic probes and 16 immune markers, it did not exhaust all commercially available metabolic probes or immunological markers. Our spectral validation protocol allows broader panel expansion. Second, we observed lower overall fluorescence intensity in multiplex staining compared to single staining. Although high MitoSOX Red concentrations have been reported to induce mitochondrial dissociation, inhibit complex IV, and suppress respiration,[39] our combined titration experiments confirmed that the MitoSOX Red concentration used in this study did not impair the mitochondrial membrane potential (Figure S6). We thus hypothesize that this phenomenon is likely attributed to high concentrations of antibodies or other components in the multiplex staining system, which may subtly reduce the staining efficiency of functional dyes, for example, via mild quenching or competition for intracellular binding sites. Third, clinical validation in the HF cohort was constrained by limited sample availability. Larger cohorts are needed to confirm clinical relevance and investigate immunometabolic pathogenesis. Fourth, although the use of 2-NBDG for glucose uptake has been widely applied, prior studies have raised concerns about its glucose transporter (GLUT)-independent uptake (a property that may introduce uncertainty into glucose utilization quantification); we, thus, incorporated 2-DG as a negative control to confirm the 2-NBDG signal could partially reflect glucose uptake in our experimental setting, but this verification remains preliminary, and future work should prioritize exploring more robust probes (e.g., radiolabeled glucose analogs or GLUT-specific fluorescent tracers) to enhance the accuracy of glucose metabolism assessments and strengthen support for our conclusions. Last, while the methodology shows synergistic potential for integration with single-cell transcriptomics and metabolomics— enabling multi-dimensional validation of cellular metabolic states—empirical benchmarking against other flow-based metabolic assays remains necessary.

RESOURCE AVAILABILITY

Lead contact

Further information and requests for resources and reagents should be directed to and will be fulfilled by the lead contact, Jie Zhang (zhangjie90905138@bjmu.edu.cn).

Materials availability

This study did not generate new unique reagents.

Data and code availability

- The data generated by this study will be shared by the lead contact upon request.
- This paper does not report original code.
- Any additional information required to reanalyze the data reported in this paper is available from the lead contact upon request.

ACKNOWLEDGMENTS

The authors acknowledge the contribution of all investigators at the participating study sites. This work was supported by the general program of National Natural Science Foundation of China (82473497) and youth programs of National Natural Science Foundation of China (82203433, 81901570), Peking University Third Hospital Talent Program (BYSY2022047, BYSY2022070), and Peking University Medicine Sailing Program for Young

Scholar's Scientific & Technological Innovation, the Fundamental Research Funds for the Central Universities (BMU2024YFJHMX006).

AUTHOR CONTRIBUTIONS

L.X., X.L., J.Y., J.R., and H.L. contributed to the concept development and study design; Y.B., Y.W., Z.G., Z.L., T.Z., and Y.L. performed the laboratory studies; Y.F. and H.L. collected the clinical samples; J.Z., Y.B., Y.W., Y.L., and D.L. contributed to data analysis, figure preparation, and drafting of the manuscript. All authors have read and approved the final manuscript.

DECLARATION OF INTERESTS

The authors declare no competing interests.

STAR★METHODS

Detailed methods are provided in the online version of this paper and include the following:

- KEY RESOURCES TABLE
- EXPERIMENTAL MODEL AND STUDY PARTICIPANT DETAILS
  - Human peripheral blood samples
  - Cancer cell line
- METHOD DETAILS
  - Isolation, cryopreservation and thawing of PBMCs
  - Establishment of fluorescence probe spectrums
  - Compensation beads staining
  - Mixed probe staining
  - Surface staining combined with probes staining
- QUANTIFICATION AND STATISTICAL ANALYSIS
  - Flow cytometry data analysis
  - Statistical analysis

SUPPLEMENTAL INFORMATION

Supplemental information can be found online at https://doi.org/10.1016/j.crmeth.2026.101330.

Received: June 26, 2025
Revised: October 20, 2025
Accepted: January 22, 2026
Published: March 16, 2026

REFERENCES

1. Finley, L.W.S. (2023). What is cancer metabolism? Cell *186*, 1670–1688. https://doi.org/10.1016/j.cell.2023.01.038.
2. Liu, H., Wang, S., Wang, J., Guo, X., Song, Y., Fu, K., Gao, Z., Liu, D., He, W., and Yang, L.L. (2025). Energy metabolism in health and diseases. Signal Transduct. Targeted Ther. *10*, 69. https://doi.org/10.1038/s41392-025-02141-x.
3. Murphy, M.P., and O'Neill, L.A.J. (2024). A break in mitochondrial endosymbiosis as a basis for inflammatory diseases. Nature *626*, 271–279. https://doi.org/10.1038/s41586-023-06866-z.
4. Tufail, M., Jiang, C.H., and Li, N. (2024). Altered metabolism in cancer: insights into energy pathways and therapeutic targets. Mol. Cancer *23*, 203. https://doi.org/10.1186/s12943-024-02119-3.
5. Kaymak, I., Williams, K.S., Cantor, J.R., and Jones, R.G. (2021). Immunometabolic Interplay in the Tumor Microenvironment. Cancer Cell *39*, 28–37. https://doi.org/10.1016/j.ccell.2020.09.004.
6. DeBerardinis, R.J., and Keshari, K.R. (2022). Metabolic analysis as a driver for discovery, diagnosis, and therapy. Cell *185*, 2678–2689. https://doi.org/10.1016/j.cell.2022.06.029.
7. Shi, Y., Zhang, H., and Miao, C. (2025). Metabolic reprogram and T cell differentiation in inflammation: current evidence and future perspectives. Cell Death Discov. *11*, 123. https://doi.org/10.1038/s41420-025-02403-1.
8. Zhang, J., Nuebel, E., Wisidagama, D.R.R., Setoguchi, K., Hong, J.S., Van Horn, C.M., Imam, S.S., Vergnes, L., Malone, C.S., Koehler, C.M., and Teitell, M.A. (2012). Measuring energy metabolism in cultured cells, including human pluripotent stem cells and differentiated cells. Nat. Protoc. *7*, 1068–1085. https://doi.org/10.1038/nprot.2012.048.
9. Palmer, A., Phapale, P., Chernyavsky, I., Lavigne, R., Fay, D., Tarasov, A., Kovalev, V., Fuchser, J., Nikolenko, S., Pineau, C., et al. (2017). FDR-controlled metabolite annotation for high-resolution imaging mass spectrometry. Nat. Methods *14*, 57–60. https://doi.org/10.1038/nmeth.4072.
10. Miller, A., Nagy, C., Knapp, B., Laengle, J., Ponweiser, E., Groeger, M., Starkl, P., Bergmann, M., Wagner, O., and Haschemi, A. (2017). Exploring Metabolic Configurations of Single Cells within Complex Tissue Microenvironments. Cell Metab. *26*, 788–800.e6. https://doi.org/10.1016/j.cmet.2017.08.014.
11. Liang, K.X., Chen, A., Kristiansen, C.K., and Bindoff, L.A. (2021). Flow Cytometric Analysis of Multiple Mitochondrial Parameters in Human Induced Pluripotent Stem Cells and Their Neural and Glial Derivatives. J. Vis. Exp. https://doi.org/10.3791/63116.
12. Zeng, Q., Lv, C., Qi, L., Wang, Y., Hao, S., Li, G., Sun, H., Du, L., Li, J., Wang, C., et al. (2024). Sodium selenite inhibits cervical cancer progression via ROS-mediated suppression of glucose metabolic reprogramming. Life Sci. *357*, 123109. https://doi.org/10.1016/j.lfs.2024.123109.
13. Zeng, Y., He, Y., Wang, L., Xu, H., Zhang, Q., Wang, Y., Zhang, J., and Wang, L. (2024). Dihydroquercetin improves experimental acute liver failure by targeting ferroptosis and mitochondria-mediated apoptosis through the SIRT1/p53 axis. Phytomedicine *128*, 155533. https://doi.org/10.1016/j.phymed.2024.155533.
14. Matschke, J., Wiebeck, E., Hurst, S., Rudner, J., and Jendrossek, V. (2016). Role of SGK1 for fatty acid uptake, cell survival and radioresistance of NCI-H460 lung cancer cells exposed to acute or chronic cycling severe hypoxia. Radiat. Oncol. *11*, 75. https://doi.org/10.1186/s13014-016-0647-1.
15. Konecny, A.J., Mage, P.L., Tyznik, A.J., Prlic, M., and Mair, F. (2024). OMIP-102: 50-color phenotyping of the human immune system with in-depth assessment of T cells and dendritic cells. Cytometry. A. *105*, 430–436. https://doi.org/10.1002/cyto.a.24841.
16. Brandi, J., Wiethe, C., Riehn, M., and Jacobs, T. (2023). OMIP-93: A 41-color high parameter panel to characterize various co-inhibitory molecules and their ligands in the lymphoid and myeloid compartment in mice. Cytometry. A. *103*, 624–630. https://doi.org/10.1002/cyto.a.24740.
17. Waaijer, L.A., van Cranenbroek, B., and Koenen, H.J.P.M. (2025). OMIP-112: 42-Parameter (40-Color) Spectral Flow Cytometry Panel for Comprehensive Immunophenotyping of Human Peripheral Blood Leukocytes. Cytometry. A. *107*, 226–232. https://doi.org/10.1002/cyto.a.24927.
18. Stanford, K.R., Hadley, S.H., Barannikov, I., Ajmo, J.M., Bahia, P.K., and Taylor-Clark, T.E. (2019). Antimycin A-induced mitochondrial dysfunction activates vagal sensory neurons via ROS-dependent activation of TRPA1 and ROS-independent activation of TRPV1. Brain Res. *1715*, 94–105. https://doi.org/10.1016/j.brainres.2019.03.029.
19. Sun, H., Li, X., Chen, X., Xiong, Y., Cao, Y., and Wang, Z. (2022). Drp1 activates ROS/HIF-1alpha/EZH2 and triggers mitochondrial fragmentation to deteriorate hypercalcemia-associated neuronal injury in mouse model of chronic kidney disease. J. Neuroinflammation *19*, 213. https://doi.org/10.1186/s12974-022-02542-7.
20. Zhou, B., Wang, D.D.H., Qiu, Y., Airhart, S., Liu, Y., Stempien-Otero, A., O'Brien, K.D., and Tian, R. (2020). Boosting NAD level suppresses inflammatory activation of PBMCs in heart failure. J. Clin. Investig. *130*, 6054–6063. https://doi.org/10.1172/JCI138538.
21. Ma, S., Ming, Y., Wu, J., and Cui, G. (2024). Cellular metabolism regulates the differentiation and function of T-cell subsets. Cell. Mol. Immunol. *21*, 419–435. https://doi.org/10.1038/s41423-024-01148-8.

22. Reina-Campos, M., Scharping, N.E., and Goldrath, A.W. (2021). CD8(+) T cell metabolism in infection and cancer. Nat. Rev. Immunol. *21*, 718–738. https://doi.org/10.1038/s41577-021-00537-8.
23. Murphy, M.P., Bayir, H., Belousov, V., Chang, C.J., Davies, K.J.A., Davies, M.J., Dick, T.P., Finkel, T., Forman, H.J., Janssen-Heininger, Y., et al. (2022). Guidelines for measuring reactive oxygen species and oxidative damage in cells and in vivo. Nat. Metab. *4*, 651–662. https://doi.org/10.1038/s42255-022-00591-z.
24. Bierling, T.E.H., Gumann, A., Ottmann, S.R., Schulz, S.R., Weckwerth, L., Thomas, J., Gessner, A., Wichert, M., Kuwert, F., Rost, F., et al. (2024). GLUT1-mediated glucose import in B cells is critical for anaplerotic balance and humoral immunity. Cell Rep. *43*, 113739. https://doi.org/10.1016/j.celrep.2024.113739.
25. Brookens, S.K., Cho, S.H., Paik, Y., Meyer, K., Raybuck, A.L., Park, C., Greenwood, D.L., Rathmell, J.C., and Boothby, M.R. (2024). Plasma Cell Differentiation, Antibody Quality, and Initial Germinal Center B Cell Population Depend on Glucose Influx Rate. J. Immunol. *212*, 43–56. https://doi.org/10.4049/jimmunol.2200756.
26. Desdin-Mico, G., Soto-Heredero, G., Aranda, J.F., Oller, J., Carrasco, E., Gabande-Rodriguez, E., Blanco, E.M., Alfranca, A., Cusso, L., Desco, M., et al. (2020). T cells with dysfunctional mitochondria induce multimorbidity and premature senescence. Science *368*, 1371–1376. https://doi.org/10.1126/science.aax0860.
27. Zhang, J., He, T., Xue, L., and Guo, H. (2021). Senescent T cells: a potential biomarker and target for cancer therapy. EBioMedicine *68*, 103409. https://doi.org/10.1016/j.ebiom.2021.103409.
28. You, Z., and Chi, H. (2023). Lipid metabolism in dendritic cell biology. Immunol. Rev. *317*, 137–151. https://doi.org/10.1111/imr.13215.
29. Li, F., Liu, H., Zhang, D., Ma, Y., and Zhu, B. (2022). Metabolic plasticity and regulation of T cell exhaustion. Immunology *167*, 482–494. https://doi.org/10.1111/imm.13575.
30. Sciarretta, S., Forte, M., Frati, G., and Sadoshima, J. (2018). New Insights Into the Role of mTOR Signaling in the Cardiovascular System. Circ. Res. *122*, 489–505. https://doi.org/10.1161/CIRCRESAHA.117.311147.
31. Wu, W., Jin, Q., Östlund, C., Tanji, K., Shin, J.Y., Han, J., Leu, C.S., Kushner, J., and Worman, H.J. (2024). mTOR Inhibition Prolongs Survival and Has Beneficial Effects on Heart Function After Onset of Lamin A/C Gene Mutation Cardiomyopathy in Mice. Circ. Heart Fail. *17*, e011110. https://doi.org/10.1161/CIRCHEARTFAILURE.123.011110.
32. Chen, Y., Xu, Z., Sun, H., Ouyang, X., Han, Y., Yu, H., Wu, N., Xie, Y., and Su, B. (2023). Regulation of CD8(+) T memory and exhaustion by the mTOR signals. Cell. Mol. Immunol. *20*, 1023–1039. https://doi.org/10.1038/s41423-023-01064-3.
33. Schiattarella, G.G., Alcaide, P., Condorelli, G., Gillette, T.G., Heymans, S., Jones, E.A.V., Kallikourdis, M., Lichtman, A., Marelli-Berg, F., Shah, S., et al. (2022). Immunometabolic Mechanisms of Heart Failure with Preserved Ejection Fraction. Nat. Cardiovasc. Res. *1*, 211–222. https://doi.org/10.1038/s44161-022-00032-w.
34. Abir, A.H., Weckwerth, L., Wilhelm, A., Thomas, J., Reichardt, C.M., Munoz, L., Völkl, S., Appelt, U., Mroz, M., Niesner, R., et al. (2024). Metabolic profiling of single cells by exploiting NADH and FAD fluorescence via flow cytometry. Mol. Metabol. *87*, 101981. https://doi.org/10.1016/j.molmet.2024.101981.
35. Arguello, R.J., Combes, A.J., Char, R., Gigan, J.P., Baaziz, A.I., Bousiquot, E., Camosseto, V., Samad, B., Tsui, J., Yan, P., et al. (2020). SCENITH: A Flow Cytometry-Based Method to Functionally Profile Energy Metabolism with Single-Cell Resolution. Cell Metab. *32*, 1063–1075.e67. https://doi.org/10.1016/j.cmet.2020.11.007.
36. Ahl, P.J., Hopkins, R.A., Xiang, W.W., Au, B., Kaliaperumal, N., Fairhurst, A.M., and Connolly, J.E. (2020). Met-Flow, a strategy for single-cell metabolic analysis highlights dynamic changes in immune subpopulations. Commun. Biol. *3*, 305. https://doi.org/10.1038/s42003-020-1027-9.
37. Fernandez-Garcia, J., Franco, F., Parik, S., Altea-Manzano, P., Pane, A.A., Broekaert, D., van Elsen, J., Di Conza, G., Vermeire, I., Schalley, T., et al. (2022). CD8(+) T cell metabolic rewiring defined by scRNA-seq identifies a critical role of ASNS expression dynamics in T cell differentiation. Cell Rep. *41*, 111639. https://doi.org/10.1016/j.celrep.2022.111639.
38. Wang, Z., Ge, S., Liao, T., Yuan, M., Qian, W., Chen, Q., Liang, W., Cheng, X., Zhou, Q., Ju, Z., et al. (2025). Integrative single-cell metabolomics and phenotypic profiling reveals metabolic heterogeneity of cellular oxidation and senescence. Nat. Commun. *16*, 2740. https://doi.org/10.1038/s41467-025-57992-3.
39. Roelofs, B.A., Ge, S.X., Studlack, P.E., and Polster, B.M. (2015). Low micromolar concentrations of the superoxide probe MitoSOX uncouple neural mitochondria and inhibit complex IV. Free Radic. Biol. Med. *86*, 250–258. https://doi.org/10.1016/j.freeradbiomed.2015.05.032.

STAR★METHODS

KEY RESOURCES TABLE

REAGENT or RESOURCE	SOURCE	IDENTIFIER
Chemicals, peptides, and recombinant proteins		
BODIPY C12	Invitrogen	Cat# D3822
BODIPY C16	Invitrogen	Cat# D3821
MitoTracker Green	Invitrogen	Cat# M7514
H2-DCFDA	Invitrogen	Cat# D399
NBD-Cholesterol (NBD)	Invitrogen	Cat# N1148
2-NBDG	Invitrogen	Cat# N13195
pHrodo Red	Invitrogen	Cat# P35372
DHE	Invitrogen	Cat# D11347
MitoSOX Red	Invitrogen	Cat# M36008
TMRE	Invitrogen	Cat# T669
MitoTracker Deep Red	Invitrogen	Cat# M22426
Antibodies		
anti-human CD3-PE-Fire640; Clone:SK7	Biolegend	Cat# 344859; RRID : AB_2860896
anti-human CD8-APC-Fire750; Clone:SK1	Biolegend	Cat# 344746; RRID: AB_2572095
anti-human CD4-Brilliant Ultraviolet 737; Clone:SK3	BD Biosciences	Cat# 612749; RRID: AB_2870080
anti-human CD45-Brilliant Ultraviolet 805; Clone: HI30	BD Biosciences	Cat# 612892; RRID: AB_2870179
anti-human CD56-BrilliantUltraviolet 496; Clone: NCAM16.2	BD Biosciences	Cat# 750479; RRID: AB_2874638
anti-human CD16-Brilliant Violet 421; Clone: B73.1	Biolegend	Cat# 360723; RRID: AB_2616913
anti-human NKG2C-PE; Clone: S19005E	Biolegend	Cat# 375003; RRID: AB_2888871
anti-human IgD-Brilliant Violet 480; Clone: IA6-2	BD Biosciences	Cat# 566187; RRID: AB_2739536
anti-human CD27-APC-Fire810; Clone: 0323	Biolegend	Cat# 302863; RRID: AB_2894450
anti-human CD19-RealBlue 744; Clone: SJ25C1	BD Biosciences	Cat# 570469; RRID: AB_3685761
anti-human CCR7-Brilliant Violet 711; Clone: G043H7	Biolegend	Cat# 353227; RRID: AB_11219587
anti-human CD45RA-Alexa Flour 700; Clone: HI100	Biolegend	Cat# 304119; RRID: AB_493762
anti-human CD25-APC; Clone: BC96	Biolegend	Cat# 302609; RRID: AB_314279
anti-human CD127-Spark NIR685; Clone: A109D5	Biolegend	Cat# 351367; RRID: AB_2890778
anti-human CD57-Pacific Blue; Clone: HNK-1	Biolegend	Cat# 359607; RRID: AB_2562458
anti-human CD28-PE-Cy7; Clone:CD28.2	Biolegend	Cat# 302926; RRID: AB_10644005
Viability-Zombie NIR	Biolegend	Cat# 423105
Biological samples		
Peripheral blood from healthy donors	Peking University Third Hospital	N/A
Peripheral blood from patients with heart failure	Peking University Third Hospital	N/A
Chemicals, peptides, and recombinant proteins		
Rot/AA	Aligent Technologies	Cat# 103344-100
Phosphate Buffered Saline (PBS)	Cytiva	Cat# SH30256.01
Fetal Bovine Serum (FBS)	GIBCO	Cat# 10099141
RPMI-1640 Medium	Cytiva	Cat# SH30027.01
Dulbecco’s Modified Eagle’s Medium (DMEM)	GIBCO	Cat# 6124597
UltraComp eBeads Plus	Invitrogen	Cat# 01-3333-42
AlignCheck	SONY	Cat# AE700510
Ficoll–PaqueTM PLUS	Cytiva	Cat# 17-5446-02
BD Horizon Brilliant Stain Buffer	BD Biosciences	Cat# 566349

(*Continued on next page*)

Continued

REAGENT or RESOURCE	SOURCE	IDENTIFIER
Penicillin- streptomycin	BD Biosciences	Cat# 15140122
Experimental models: Cell lines		
Human: A2780	Cell Resources Center of Peking Union Medical College (Beijing, China)	N/A
Human: MDA-MB-231	Cell Resources Center of Peking Union Medical College (Beijing, China)	N/A
Software and algorithms		
Prism10.4.1	GraphPad	N/A
FlowJo10.10.0	BD Biosciences	N/A
Kaluza	Beckman	N/A
Other		
ID7000	SONY	N/A

EXPERIMENTAL MODEL AND STUDY PARTICIPANT DETAILS

Human peripheral blood samples

This study included newly diagnosed heart failure patients. Peripheral blood (0.5 mL) was collected from 6 healthy subjects and 7 heart failure patients in 2023–2024 at Peking University Third Hospital (Beijing, China), and stored in Ethylenediaminetetraacetic acid (EDTA) anti-coagulant tubes (BD, USA). All sampling and experimental steps in this study were approved by the Ethics Committee of Peking University Third Hospital (License No. M2022108).

Cancer cell line

Human ovarian cancer cell line A2780 and Human breast cancer cell line MDA-MB-231 were purchased from the Cell Resources Center of Peking Union Medical College (Beijing, China). A2780 were grown in RPMI-1640 (Cytiva, Cat # SH30027.01) and MDA-MB-231 were grown in DMEM (GIBCO, Cat # 6124597), which all supplemented with 10% fetal bovine serum (FBS, GIBCO, Cat # 10099141), 50 IU/mL penicillin, and 50 mg/mL streptomycin (GIBCO, Cat # 15140122). All cells were cultured at 37°C in a humidified incubator in the presence of 5% CO_2 and 20% O_2.

The cells were validated by STR analysis at the Cell Resources Center of Peking Union Medical College and regularly tested for mycoplasma contamination (culture testing).

METHOD DETAILS

Isolation, cryopreservation and thawing of PBMCs

Peripheral blood was centrifuged at 2000 × g for 10 min at 4°C. PBMC were isolated by density gradient centrifugation with Ficoll–Paque PLUS (Cytiva, Cat#17-5446-02) and PBS at a ratio of 1:1.5. Samples were centrifuged at 400 × g for 20 min without brake at 20°C. Cells were harvested and washed twice with PBS at 500 × g for 5 min and counted manually. The residual PBMC were cryopreserved in a solution consisting of 10% dimethyl sulfoxide (DMSO) and 90% fetal bovine serum (FBS, GIBCO, Cat # 10099141), and were subsequently stored in liquid nitrogen following a 24-h period at −80°C.

The cryopreserved PBMC were rapidly thawed in a 37°C water bath with gentle agitation. Immediately after thawing, the cell suspension was transferred to a 15 mL centrifuge tube containing pre-warmed RPMI-1640 medium (Hyclone, USA). The cells were then collected by centrifugation at 500*g* for 5 min. After discarding the supernatant, the cell pellet was resuspended and washed once with phosphate-buffered saline (PBS). Finally, the washed cells were ready for subsequent staining and analysis.

Establishment of fluorescence probe spectrums

For fluorescent probes staining, freshly-isolated A2780 (2×10^5 per condition) were stained in a volume of 100 µL PBS (Cytiva, Cat # SH30256.01) with one of the fluorescent probes for 30 min at 37°C. Cells were washed and then acquired by full spectrum flow cytometry (SONY ID7000). The working concentration of all fluorescent probes are listed in Table 1. Negative and positive controls confirm the specificity of the probes used in our study (Figure S1D).

Compensation beads staining

For compensation beads (Invitrogen, Cat#01-3333-42) staining, 1 drop of compensation beads were stained with antibodies conjugated with FITC, PE, Alexa Flour 488, APC or Alexa Flour 647 for 15 min under dark room temperature conditions. After wash, beads were re-suspended with 200 µL PBS.

For interprobe resolution or probe-fluorescein resolution, the stained A2780 cells or the stained beads were mixed together and then detected by SONY ID7000.

Mixed probe staining

For A2780 staining, freshly-digested A2780 (2×10^5) were incubated 50 μL PBS in 0.5 μM Rot/AA (Aligent Technologies, Cat# 103344-100) for 30 min at 37°C. Subsequently, cells were washed with 1mL PBS and incubated in 100 μL PBS containing 100 nM H2-DCFDA, 3 μM MitoSOX Red and 5 nM TMRE for 30 min at 37°C. After the final wash, cells were re-suspended with PBS and the fluorescence intensity was detected by SONY ID7000. A minimum of 10,000 single cells were acquired for analysis.

Freshly-digested MDA-MB-231 and EZH2-knockdown MDA-MB-231 cells (2×10^5) were incubated in 100 μL PBS containing 100 nM H2-DCFDA, 3 μM MitoSOX Red and 5 nM TMRE for 30 min at 37°C. EZH2-knockdown MDA-MB-231 cells established via lentiviral shRNA delivery. Cells were re-suspended with PBS and the fluorescence intensity was detected by SONY ID7000 after wash. A minimum of 10,000 single cells were acquired for analysis. Since short-term storage (Figure S2D), fixation (Figure S2E), and stained cell number (Figure S2C) can impact metabolic probe MFI; thus, we recommend on-instrument analysis immediately post-staining to minimize potential minor MFI fluctuations and ensure optimal experimental accuracy.

Surface staining combined with probes staining

Freshly-isolated or cryopreserved PBMC (1×10^6) were stained with surface antibodies in a total volume of 50 μL BV staining buffer (BD Biosciences, Cat# 566349) for 30 min at room temperature in the dark. Subsequently, cells were washed with 1mL PBS and incubated in 100 μL PBS containing 1 μM BODIPY C12, 100 μM 2-NBDG, 3μM MitoSOX Red or 5nM TMRE for 30min at 37°C. In addition, a tube of cells was stained with all four probes. After proper incubation and washing, cells were further stained in 50 μL PBS with Zombie NIR Fixable Live/Dead cell stain for 10 min at room temperature. Cells were re-suspended with PBS for data acquisition by SONY ID7000 after the final wash. A minimum of 20,000 $CD45^+$ T cells were acquired for analysis. All surface antibodies are listed in Table S1.

QUANTIFICATION AND STATISTICAL ANALYSIS

Flow cytometry data analysis

Data were analyzed using FlowJo 10.10.0 (BD Biosciences) and Prism 10.4.1 (Graphpad Software). Gating strategy was shown in Figure S4C. Breifly, target cells were gated based on forward scatter (FSC) and side scatter (SSC) profiles to isolate the cell population of interest. Next, doublets or adherent cells were excluded using forward scatter area (FSC-A) versus forward scatter height (FSC-H) plots. Subsequently, live cells were identified as Zombie NIR^- cells, and immune cells were further gated as $CD45^+$ cells within the live cell population. We then performed unsupervised clustering on $CD45^+$ cells with embedded plug-ins (t-SNE, X-Shift, FLOWSOM). All fluorophores and metabolic probes were used for t-SNE analysis. tSNE parameters were set as follows: iterations (1000), perplexity (30), and eta (20972). For immune cell subset analysis, all surface markers were analyzed using the X-Shift method. For metabolic subset analysis, 4 metabolic probes were selected for analysis via FLOWSOM. Heatmaps were generated using Prism 10.4.1 (Graphpad Software).

Statistical analysis

Statistical analysis of results was carried out using Prism 10.4.1 (GraphPad Software). Correlation analysis were performed using Pearson correlation. Statistical testing was two-sided and *p*-values <0.05 were considered statistically significant.

Cell Reports Methods, Volume *6*

Supplemental information

Application of spectral flow cytometry for comprehensive detection of immune metabolism in patient-derived microsamples

Yang Bai, Yuqing Wang, Yicheng Fu, Zhengyang Guo, Zhaoyuan Liang, Liu Yang, Jiawei Ribaudo, Dan Liu, Yanfang Li, Ting Zhang, Lixiang Xue, Jianling Yang, Huilin Liu, Xianlong Li, and Jie Zhang

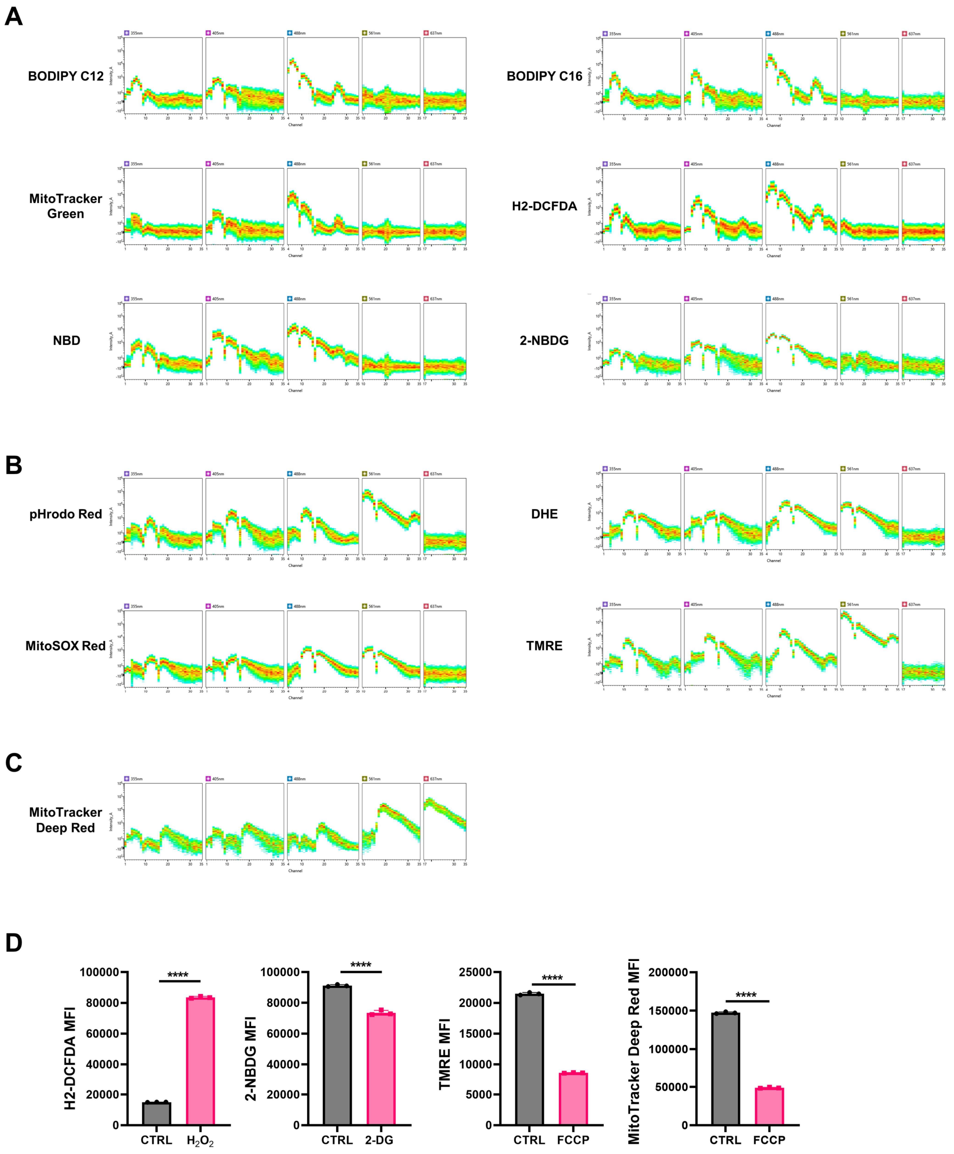

Figure S1. Establishment of an 11-probe spectral reference library, related to Figure 1 and STAR Methods.

Spectral profiles of metabolic probes for channel-specific reference generation: **(A)** FITC channel (6 probes). **(B)** PE channel (4 probes). **(C)** APC channel (1 probe). (**D**) Positive controls: H_2O_2 for H2-DCFDA. Negative controls: 2-deoxy-D-glucose (2-DG) for 2-NBDG; FCCP for TMRE and MitoTracker Deep Red (n=3). ****, $p < 0.0001$.

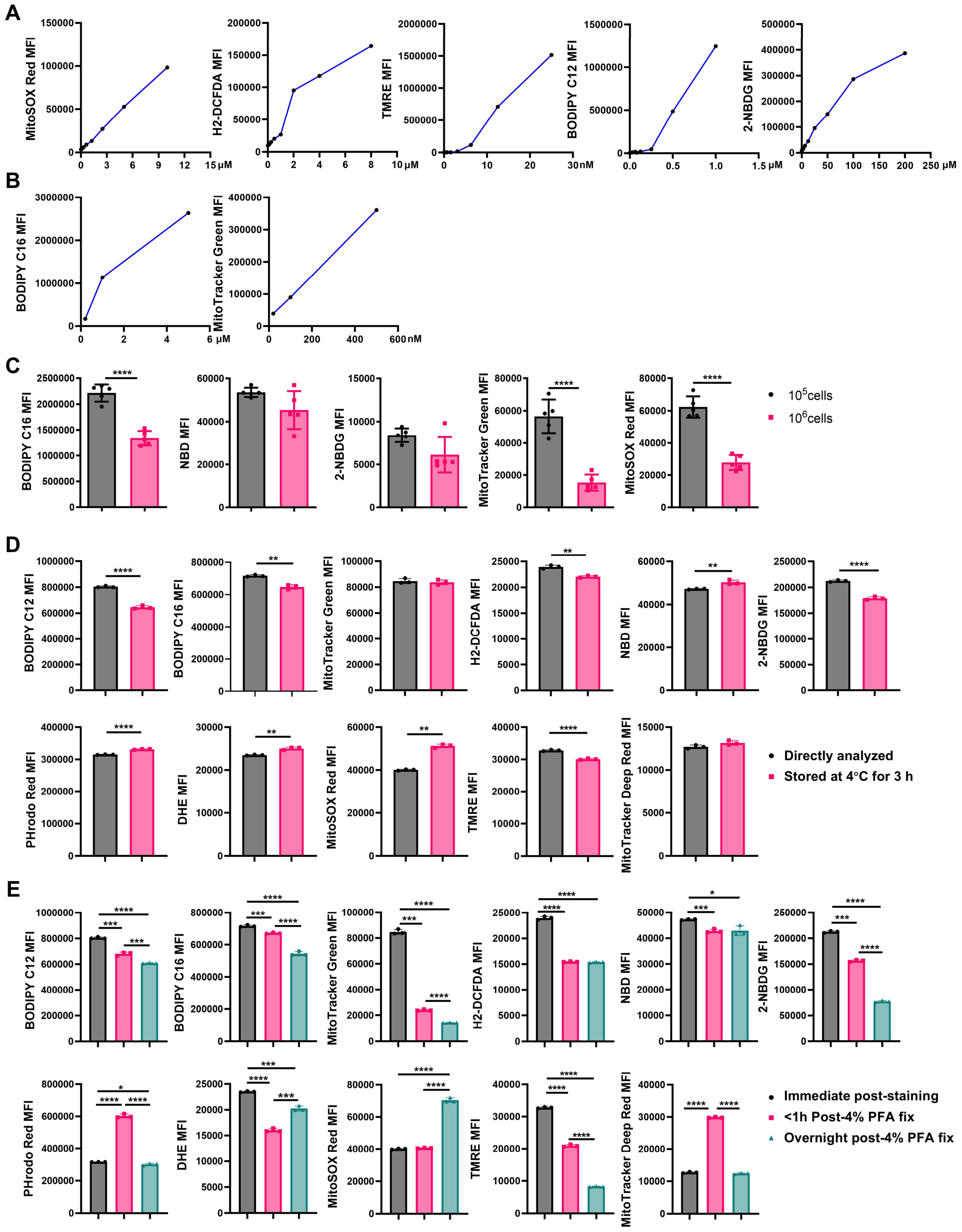

Figure S2. Characterization of metabolic probe titration and staining stability, related to Figure 1 and STAR Methods.

Titration curves of staining probes across 10 concentrations **(A)** and 3 concentrations **(B)** within instrument detection range. **(C)** Mean fluorescence intensity (MFI) of 10^5 and 10^6 A2780 cells stained with 5 distinct metabolic probes (n=5). **(D-E)** Evaluation of staining stability. A2780 cells were stained with 11 distinct metabolic probes. **(D)** MFI of cells analyzed immediately after staining was compared to that of cells stored at 4°C in the dark for 3 hours (n=3). **(E)** MFI comparison across three conditions: immediate analysis; analysis within 1 h post 4% paraformaldehyde (PFA) fixation; and analysis after overnight storage (n=3). *, $p < 0.05$; **, $p < 0.01$; ***, $p < 0.001$; ****, $p < 0.0001$.

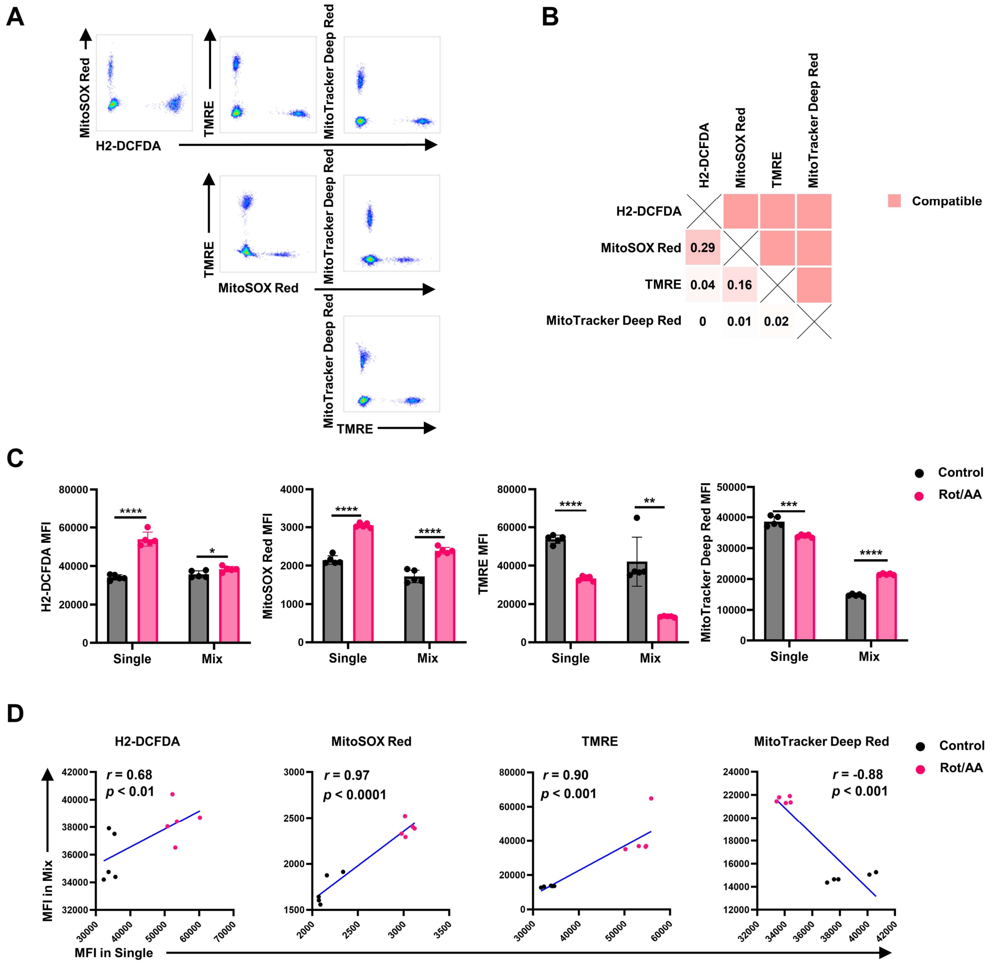

Figure S3. Validation of four metabolic probes for simultaneous assessment of mitochondrial activity and oxidative stress, related to Figure 3.

(A) Representative flow cytometry density plots demonstrating spectral resolution of co-stained probes. **(B)** Validation matrix: Lower left quadrant - Computational spectral similarity by FluoroFinder; Upper right quadrant - Empirical resolvability determination (Red: resolvable pairs). **(C)** Mean fluorescence intensity (MFI; mean ± SD) of individual probes in control versus Rotenone/Antimycin A (Rot/AA)-treated groups (n=5). **(D)** Correlation analysis of MFI between single-stain and multiplexed conditions across probes (Spearman r). *, $p < 0.05$; **, $p < 0.01$; ***, $p < 0.001$; ****, $p < 0.0001$.

A

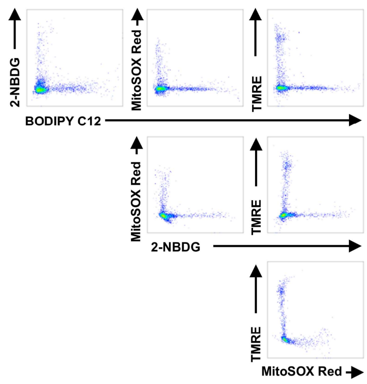

B

	BODIPY FL *	2-NBDG *	MitoSOX Red *	TMRE *	PE-Fire 640	APC-Fire 750	Brilliant Ultraviolet 737	Brilliant Ultraviolet 805	Brilliant Ultraviolet 496	Brilliant Violet 421	PE	Brilliant Violet 480	APC-Fire 810	RealBlue 744	Brilliant Violet 711	Alexa Fluor 700 *	APC	Spark NIR 685	Pacific Blue	PE-Cy7	Zombie NIR
BODIPY FL *	1.0	0.69	0.13	0.01	0.0	0.0	0.0	0.01	0.13	0.01	0.03	0.05	0.0	0.03	0.0	0.0	0.0	0.0	0.02	0.0	0.0
2-NBDG *	0.69	1.0	0.63	0.07	0.06	0.0	0.01	0.02	0.23	0.03	0.25	0.16	0.01	0.04	0.01	0.0	0.01	0.0	0.07	0.02	0.0
MitoSOX Red *	0.13	0.63	1.0	0.16	0.24	0.0	0.04	0.01	0.1	0.01	0.47	0.05	0.02	0.06	0.04	0.0	0.05	0.02	0.02	0.03	0.01
TMRE *	0.01	0.07	0.16	1.0	0.19	0.0	0.0	0.0	0.0	0.0	0.86	0.0	0.01	0.0	0.0	0.0	0.03	0.01	0.0	0.05	0.0
PE-Fire 640	0.0	0.06	0.24	0.19	1.0	0.06	0.07	0.01	0.01	0.0	0.16	0.01	0.11	0.1	0.17	0.14	0.49	0.24	0.0	0.1	0.1
APC-Fire 750	0.0	0.0	0.0	0.0	0.06	1.0	0.18	0.28	0.0	0.0	0.0	0.01	0.78	0.04	0.16	0.18	0.11	0.19	0.0	0.37	0.37
Brilliant Ultraviolet 737	0.0	0.01	0.04	0.0	0.07	0.18	1.0	0.32	0.05	0.02	0.01	0.01	0.12	0.16	0.31	0.18	0.1	0.14	0.01	0.11	0.32
Brilliant Ultraviolet 805	0.01	0.02	0.01	0.0	0.01	0.28	0.32	1.0	0.14	0.04	0.01	0.02	0.28	0.05	0.05	0.02	0.02	0.03	0.01	0.13	0.04
Brilliant Ultraviolet 496	0.13	0.23	0.1	0.0	0.01	0.0	0.05	0.14	1.0	0.1	0.03	0.43	0.03	0.01	0.02	0.0	0.01	0.01	0.22	0.01	0.0
Brilliant Violet 421	0.01	0.03	0.01	0.0	0.0	0.0	0.02	0.04	0.1	1.0	0.0	0.25	0.02	0.0	0.17	0.0	0.0	0.0	0.69	0.0	0.0
PE	0.03	0.25	0.47	0.86	0.16	0.0	0.01	0.01	0.03	0.0	1.0	0.03	0.01	0.01	0.01	0.0	0.03	0.01	0.01	0.06	0.0
Brilliant Violet 480	0.05	0.16	0.05	0.0	0.01	0.01	0.01	0.02	0.43	0.25	0.03	1.0	0.03	0.0	0.05	0.0	0.01	0.01	0.67	0.01	0.0
APC-Fire 810	0.0	0.01	0.02	0.01	0.11	0.78	0.12	0.28	0.03	0.02	0.01	0.03	1.0	0.03	0.15	0.16	0.21	0.21	0.03	0.25	0.14
RealBlue 744	0.03	0.04	0.06	0.0	0.1	0.04	0.16	0.05	0.01	0.0	0.01	0.0	0.03	1.0	0.05	0.02	0.01	0.02	0.0	0.34	0.19
Brilliant Violet 711	0.0	0.01	0.04	0.0	0.17	0.16	0.31	0.05	0.02	0.17	0.01	0.05	0.15	0.05	1.0	0.24	0.18	0.24	0.12	0.13	0.17
Alexa Fluor 700 *	0.0	0.0	0.0	0.0	0.14	0.18	0.18	0.02	0.0	0.0	0.0	0.0	0.16	0.02	0.24	1.0	0.34	0.68	0.0	0.05	0.67
APC	0.0	0.01	0.05	0.03	0.49	0.11	0.1	0.02	0.01	0.0	0.03	0.01	0.21	0.01	0.18	0.34	1.0	0.65	0.0	0.04	0.22
Spark NIR 685	0.0	0.0	0.02	0.01	0.24	0.19	0.14	0.03	0.01	0.0	0.01	0.01	0.21	0.02	0.24	0.68	0.65	1.0	0.01	0.05	0.38
Pacific Blue	0.02	0.07	0.02	0.0	0.0	0.0	0.01	0.01	0.22	0.69	0.01	0.67	0.03	0.0	0.12	0.0	0.0	0.01	1.0	0.0	0.0
PE-Cy7	0.0	0.02	0.03	0.05	0.1	0.37	0.11	0.13	0.01	0.0	0.06	0.01	0.25	0.34	0.13	0.05	0.04	0.05	0.0	1.0	0.15
Zombie NIR	0.0	0.0	0.01	0.0	0.1	0.37	0.32	0.04	0.0	0.0	0.0	0.0	0.14	0.19	0.17	0.67	0.22	0.38	0.0	0.15	1.0

C

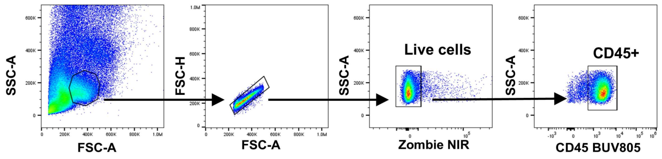

Figure S4. Validation of spectral co-resolvability in immunometabolic profiling panels, related to Figure 4 and STAR Methods.

(A) Representative flow cytometry plots demonstrating pairwise resolution of co-stained metabolic probes. **(B)** Comprehensive FluoroFinder computational matrix quantifying spectral similarity among 4 metabolic probes, 16 surface markers, and 1 viability dye. **(C)** Gating strategy.

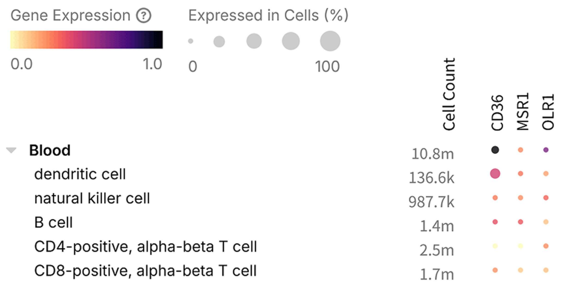

Figure S5. scRNA-seq analysis of lipid-uptake receptor expression across human peripheral blood immune subsets, related to Figure 5 and 6.

Analysis of *CD36*, *MSR1* (CD204), and *OLR1* (LOX-1) expression across functionally defined immune cell subsets using published human peripheral blood mononuclear cell (PBMC) scRNA-seq data (CZI Cellxgene platform: cellxgene.cziscience.com).

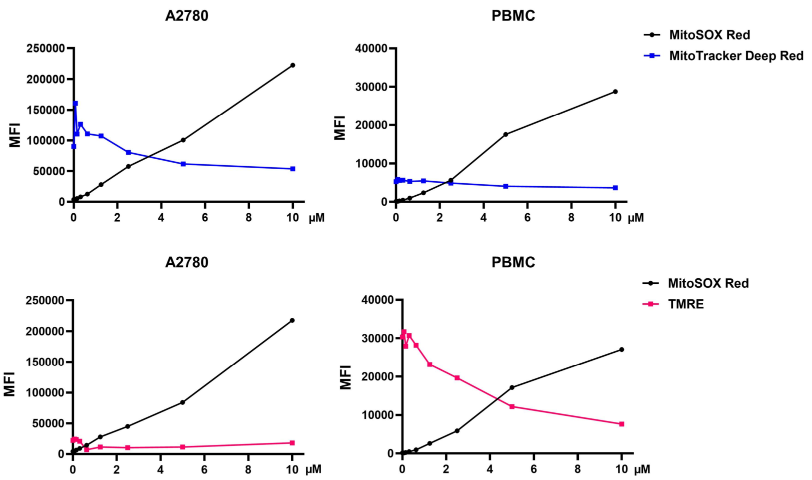

Figure S6. Effect of MitoSOX Red staining concentration on mitochondrial membrane potential, related to Figure 3 and 4.

Dose titration experiments of MitoSOX Red to assess its effect on TMRE and MitoTracker Deep Red, conducted in both A2780 cells and PBMCs.

Table S1 Antibody panel for metabolic co-staining

Specificity	Fluorochrome	Purpose	Clone	Manufacturer	Catalog NO.	Dilution of Stock
CD3	PE-Fire640	Pan T cell, NKT-Like cells	SK7	Biolegend	344859	1:100
CD8	APC-Fire750	CD8 T, NK, and NKT-Like cells	SK1	Biolegend	344746	1:100
CD4	Brilliant Ultraviolet 737	CD4 T and NKT-Like cells	SK3	BD Biosciences	612749	1:100
CD45	Brilliant Ultraviolet 805	Leukocytes	HI30	BD Biosciences	612892	1:100
CD56	Brilliant Ultraviolet 496	Pan NK cell, γδ T cell activation	NCAM16.2	BD Biosciences	750479	1:100
CD16	Brilliant Violet 421	Monocyte, NK cell, and dendritic cell differentiation	B73.1	Biolegend	630723	1:100
NKG2C	PE	NK cell differentiation	S19005E	Biolegend	375003	1:100
IgD	Brilliant Violet 480	B cell differentiation	IA6-2	BD Biosciences	566187	1:400
CD27	APC-Fire810	T and B cell differentiation	0323	Biolegend	302863	1:50
CD19	RealBlue 744	B cells	SJ25C1	BD Biosciences	570469	1:100
CCR7	Brilliant Violet 711	T cell differentiation	G043H7	Biolegend	353227	1:50
CD45RA	Alexa Flour 700	T cell and dendritic cell differentiation	HI100	Biolegend	304119	1:25
CD25	APC	Regulatory T cells	BC96	Biolegend	302609	1:50
CD127	Spark NIR685	Cytokine receptor; T cell differentiation	A109D5	Biolegend	351367	1:100
CD57	Pacific Blue	NK and CD8+ T cell immune senescence	HNK-1	Biolegend	359607	1:200
CD28	PE-Cy7	T cell and NK cell differentiation	CD28.2	Biolegend	302926	1:50
Viability	Zombie NIR	Viability		Biolegend	423105	1:200
